# Initiation at AUGUG and GUGUG sequences can lead to translation of overlapping reading frames in *E. coli*

**DOI:** 10.1093/nar/gkac1175

**Published:** 2022-12-22

**Authors:** Maximilian P Kohl, Maria Kompatscher, Nina Clementi, Lena Holl, Matthias D Erlacher

**Affiliations:** Institute of Genomics and RNomics, Biocenter, Medical University of Innsbruck, 6020 Innsbruck, Austria; Institute of Genomics and RNomics, Biocenter, Medical University of Innsbruck, 6020 Innsbruck, Austria; Institute of Genomics and RNomics, Biocenter, Medical University of Innsbruck, 6020 Innsbruck, Austria; Institute of Genomics and RNomics, Biocenter, Medical University of Innsbruck, 6020 Innsbruck, Austria; Institute of Genomics and RNomics, Biocenter, Medical University of Innsbruck, 6020 Innsbruck, Austria

## Abstract

During initiation, the ribosome is tasked to efficiently recognize open reading frames (ORFs) for accurate and fast translation of mRNAs. A critical step is start codon recognition, which is modulated by initiation factors, mRNA structure, a Shine Dalgarno (SD) sequence and the start codon itself. Within the *Escherichia coli* genome, we identified more than 50 annotated initiation sites harboring AUGUG or GUGUG sequence motifs that provide two canonical start codons, AUG and GUG, in immediate proximity. As these sites may challenge start codon recognition, we studied if and how the ribosome is accurately guided to the designated ORF, with a special focus on the SD sequence as well as adenine at the fourth coding sequence position (A4). By *in vitro* and *in vivo* experiments, we characterized key requirements for unambiguous start codon recognition, but also discovered initiation sites that lead to the translation of both overlapping reading frames. Our findings corroborate the existence of an ambiguous translation initiation mechanism, implicating a multitude of so far unrecognized ORFs and translation products in bacteria.

## Graphical Abstract

**Graphical Abstract:**
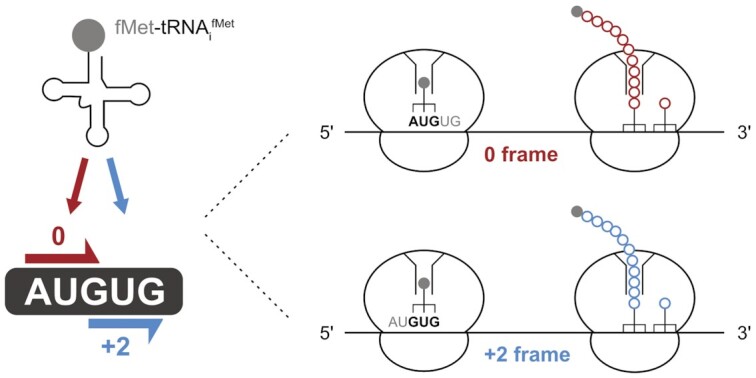
AUGUG and GUGUG initiation sequences can represent ambiguous start sites and challenge start codon selection in *E. coli*.

## INTRODUCTION

Translation initiation is an intricate process that secures the fast and accurate recognition of start codons and thereby defines the open reading frame (ORF) of an mRNA. In bacteria, translation initiation is a well-orchestrated interplay of mRNAs, 30S ribosomal subunits, the initiator tRNA (tRNA_i_^fMet^) and three initiation factors (IFs). The initiation phase of translation begins with the formation of a labile 30S pre-initiation complex (30S pre-IC), in which the start codon of the mRNA is decoded by the anticodon of the initiator tRNA_i_^fMet^ in the P-site of the small ribosomal subunit. This step is assisted by the initiation factors IF1, IF2 and IF3, which contribute to the fidelity of start codon recognition and impact the kinetics of the initiation step. Subsequently, the 30S pre-IC is stabilized in a locked conformation, referred to as 30S initiation complex (30S IC). The 30S IC is then joined by the 50S ribosomal subunit to yield the 70S initiation complex (70S IC), capable of forming the first peptide bond between formyl-methionine of the P-site bound fMet-tRNA_i_^fMet^ and the aminoacyl-tRNA located at the A-site. During this transition, the three IFs dissociate from the complex and the first EF-G-dependent translocation marks the beginning of the elongation phase of protein synthesis (1–7).

Importantly, start codon recognition itself represents a key kinetic checkpoint that governs the speed and the fidelity of the initiation step (3,4,8). However, prior to start codon recognition, it is essential that the correct translation initiation region (TIR) is selected on mRNAs harboring multiple potential ORFs. Consequently, the ribosomal binding site (RBS) has to provide the ability to recruit and position the initiation complex to the designated start codon, thereby dictating cistron specificity. In many bacterial mRNAs, this key function of the RBS is facilitated by the presence of a Shine-Dalgarno (SD) sequence motif (9,10). An SD sequence aids the recruitment of 30S subunits to the RBS by forming base pairs with the anti-SD (aSD) sequence located at the 3′ end of the 16S rRNA (9–17). A stable SD:aSD interaction not only promotes the recruitment of the initiation complex, but also positions the start codon in the vicinity of the ribosomal P-site for its decoding (18–21). To do so, SD sequences are typically separated from the start codon by an approximately 4-8 nucleotide (nt) long spacer element that does not base pair with the 16S rRNA (22).

Although a principle role of the SD:aSD interaction during initiation is well established, a large proportion of the genes expressed in *Escherichia coli* and other bacteria does not harbor a classical SD sequence (23–29). Furthermore, even leaderless mRNAs, lacking 5′ untranslated regions (UTRs), were described to be translationally competent (30–33). Consistently, a recent ribosome profiling study employing 16S-mutant ribosomes with an altered aSD sequence demonstrated that in essence all translation initiation sites could be recognized without the aid of an SD sequence in *E. coli* (29). Therefore, TIRs seem to be intrinsically hard-wired by additional mRNA features, such as their general unstructured nature and A-rich 5′ UTRs (15,29,34–44). Because the dispensable nature of SD:aSD base pairing implies that SD sequences primarily enhance 30S recruitment but are non-essential for ORF definition, the start codon itself remains as the most important element of the RBS. However, even the start codon is not strictly defined. A recent analysis of alternative start codon usage in over 60 prokaryotic genomes revealed that ∼82% of annotated sites accounted for cognate AUG start codons, while GUG and UUG were utilized by approximately ∼14% and ∼4%, respectively. Besides these three canonical start codons, even further degenerate initiation triplets were shown to provide measureable initiation efficiencies (45).

Considering the importance of clear ORF definition, the abundance of alternative start codons and the flexibility in their recognition bear peculiar implications for initiation fidelity. For example, they may imply supposedly ill-defined initiation sites, in which multiple potential start codons lie in close proximity or even overlap (e.g. AUGUG, GUGUG). Within the *E. coli* reference genome, we identified 53 genes harboring an annotated ATG or GTG start codon within a potentially ambiguous ATGTG or GTGTG sequence context. Because start codon recognition represents a rate-limiting step during initiation (3), these sequence motifs could imply significant consequences for gene expression.

The immediate proximity of the potential start codons could serve a strictly regulatory purpose. In this regard, already in 1987, Larry Gold and colleagues noted that in one of their tested reporter sequences, initiation from a GUG-starting construct was approximately 50-fold reduced within an AUGUG sequence context (46). In contrast, some well-characterized and efficiently expressed genes, e.g. the lac repressor (*lacI*), harbor such unfavorable initiation motifs, raising the question how the presumably less potent GUG codon could be efficiently selected in proximity of AUG. Therefore, the arrangement of start codon overlaps could also have only little consequence for the expression of the respective genes, if the initiation complex was precisely directed towards the designated start codon. The latter would imply that the RBS architecture, e.g. the structure, the SD-spacer composition and the annotated start codon, were sufficient to univocally define the start site.

Additionally, start codon overlaps could represent a strategy for the expression of two overlapping ORFs and thereby effectively increase the coding capacity of the genome. This hypothesis would imply that the unusual sequence arrangement could provide a mechanism to regulate the expression of genes co-dependently by partitioning of initiation complexes at competing start codons. The existence of a similar mechanism was postulated about 20 years ago in *Thermus thermophilus*, where the gene for ribosomal protein L34 (*rpmH*) was found to be fully nested within the gene for the RNase P subunit protein (*rnpA*) (47). Sharing a single translation initiation site, the two AUG start codons of the respective ORFs are only separated by a single nucleotide (5′-AUGGAUG-3′) and were demonstrated to provide the efficient expression of both genes. Although the *rnpA-rpmH* initiation site has remained the only described case in the bacterial kingdom so far, the postulated mechanism might be far more common than previously anticipated.

To understand how the immediate vicinity of two potential start codons affects ORF selection during initiation at AUGUG and GUGUG sequences, we employed different *in vitro* and *in vivo E. coli* translation systems. Thereby, we identified various prerequisites that provide the required precision to guide initiation complexes to one start codon or the other, ensuring efficient translation of the respective ORF. Whereas in most overlaps in *E. coli*, start codon recognition indeed appears to be precisely defined, some RBSs provide the ability to initiate at both start codons and thus confer the translation of two overlapping ORFs. Our findings imply that initiation at AUGUG and GUGUG sequences can lead to the expression of so far unrecognized, overlapping ORFs, effectively expanding the variability of the bacterial proteome.

## MATERIALS AND METHODS

### Plasmids and constructs

A firefly luciferase (fLUC) reporter gene was cloned downstream of a T7 promotor sequence and the RBS derived from the bacterial *ermCL* 5′ UTR into the multiple cloning site of a pUC19 vector to generate a distinct AUG reference construct (ME_in2) (48). Sequence variations were introduced using the Q5 site-directed mutagenesis kit (New England Biolabs, NEB, E0552S). This kit was also employed to introduce the native RBS of candidate genes, whereby the fLUC RBS was replaced with native initiation regions (20 nts of 5′ UTR and 15 nts of the coding sequence). The GFP plasmids for the *in vivo* studies were based on pRXGSM-sfsA (49). Initially, by use of NEBuilder™ HiFi DNA Assembly (NEB, E2621), the *sfsA* sequence upstream of sfGFP was substituted with the 5′ UTR of the reference luciferase plasmid (ME_in2) to create an analogous sfGFP reference with distinct ATG start codon (pRXG_MK1). Employing site directed mutagenesis, various RBS GFP reporter mutants were generated. All required DNA oligonucleotides were obtained from Integrated DNA Technologies (IDT). Every reporter construct was verified by Sanger sequencing (Eurofins genomics). The initiation sequences of all reporter plasmids are provided in the Supplementary Tables S1–S8.

### *In vitro* transcription

The DNA templates for *in vitro* transcriptions encoded a T7 promotor 5′ to the respective RBS. The templates for transcription were generated by PCR amplification employing the Phusion™ DNA polymerase (Thermo Fisher, F534L) and purified by use of the Monarch™ PCR purification system (NEB, T1030L). The transcripts were generated with the HiScribe™ T7 High Yield RNA Synthesis Kit (NEB, E2040S) according to the manufacturer's protocol and subsequently purified following RQ1 DNase digestion (Promega, 610A) by use of the Monarch™ RNA Cleanup Kit (NEB, T2040).

### *In vitro* translation (IVT)

S30-extract based IVTs for luciferase reporter assays were carried out using the cell-free NEBExpress™ system (NEB, E5360L) according to the manufacturer's protocol, but scaled to a final reaction volume of 6.25 μl. 30 ng of the respective reporter plasmids were used as templates and the reactions were incubated at 37°C for 45 min. 25 μl of luciferase assay substrate (Promega, E1500) were pipetted into black clear-bottom 96-well plates before 2 μl of the reactions were pipetted to the inner wall of the wells. A brief centrifugation step at 1000 rpm served to synchronize the timing of the luciferin turnover. After 3 min, the luminescent signal was determined in a ‘BMG Labtech FLUOstar Omega’ plate reader. All sets included the translation of a standard reporter plasmid (ME_in2) (Supplementary Table S1), which was used as a reference to enable the comparison between different sets of tested reporter plasmids. Control reactions were carried out without template and subtracted from all measurements. The measurements were performed in technical duplicates, which were averaged to obtain single data points of individual reactions. For all data shown, at least three independent reactions were performed.

The release factor (RF) dependence of translation was determined employing the PURExpress ***Δ***RF123 *in vitro* translation system (NEB, E6850S) according to the provided protocol. As template, 30 ng of the corresponding plasmids were added to the reactions. IVTs were performed either in presence of RF1 and RF3 or RF2 and RF3. The final volume was scaled to 6.5 μl and the reactions were incubated for 10 min at 37°C. Relative luciferase activity was assayed as described above.

For the direct detection of translation products, *in vitro* translations were carried out in the presence of ^35^S-labeled methionine and cysteine. Therefore, the recombinant PURExpress™ *in vitro* protein synthesis kit (NEB, E6800S) was used according to the manufacturer's protocol, but scaled to a final reaction volume of 6.25 μl. The reactions contained 50 ng of reporter plasmid as template and 5 μCi ^35^S-Met/Cys (Hartmann Analytic, SCIS-103). After 60 min of incubation at 37°C, Laemmli sample buffer was added to the translation reactions and the protein products were resolved by a 18% Tris-Tricine SDS PAGE (50). After the gel was dried in a slab gel dryer, it was exposed to a phosphorimaging screen overnight and subsequently scanned on a Typhoon™ FLA 9500.

### *In vivo* translation

*In vivo* fluorescence measurements were performed in principle as described by Meydan *et al.* (49). The GFP reporter plasmids were transformed into 5-alpha *E. coli* cells (NEB, C2987H) and grown overnight in 5 ml LB supplemented with 50 mg/ml kanamycin. Subsequently, the optical density of the cultures was adjusted with fresh LB/kanamycin to OD_600_ of 0.2. The cultures were grown for another 4 h at 37°C, shaking at 160 rpm. The cell density was then adjusted to OD_600_ of 0.02 in 5 ml of fresh LB/kanamycin, before GFP expression was induced by the addition of IPTG (0.1 mM f.c.). The induced cultures were incubated for 20 h at 37°C, shaking at 160 rpm, and subsequently 120 μl were transferred into clear flat-bottom 96-well plates for measurement in a ‘BMG Labtech CLARIOstar Plus’ plate reader. Prior to fluorescence measurement, plates were subjected to orbital shaking at 300 rpm for 30 s in the plate reader. Green fluorescence was measured by excitation at 485 nm and emission detection at 585 nm with a gain setting of 1250. Additionally, optical density of the samples was measured in the plate reader by absorbance at 700 nm. Fluorescent signal from 120 μl LB/kanamycin was subtracted from sample measurements, which were then normalized to optical density.

### Toeprint assays

Toeprint experiments were based on previously described protocols (49,51), but modified in several aspects. The toeprinting primer oMKtoe2 (5′-GTTCCATCTTCCAGCGGATAG-3′) was PAGE purified before labeling. For a toeprint reaction, 1 pmol of primer was labeled using 2 μCi of gamma-[^32^P]-ATP (Hartmann Analytic, SRP-501) and 1 U of T4 polynucleotide kinase (NEB, M0201S) in the buffer provided by the manufacturer. The labeling reaction was incubated for 30 min at 37°C and afterwards inactivated for 2 min at 95°C. In parallel, the *in vitro* translation reactions were carried out using the PURExpress™ *in vitro* protein synthesis kit (NEB, E6800S) according to the manufacturer's protocol. A typical reaction contained 2 μl of solution A, 1.8 μl of solution B, 50 μM retapamulin (Sigma Aldrich, CDS023386) and 200 ng RNA template in 6.25 μl total reaction volume. For control experiments without ribosomes, a specialized ***Δ***ribosome, ***Δ***RF123 IVT system was used (NEB, E6850ZZ). The samples were incubated for 30 min at 37°C. Subsequently, 2 μl of the labeling reaction were directly added to the IVTs together with 10 U of Ribolock RNase inhibitor (Thermo Fisher, EO0381). After 2 min at 37°C, the reactions were incubated on ice for 5 min to allow hybridization of the primer. For primer extension, the reactions were supplied with a dNTP mix (0.32 mM f.c. each) and 15 U of AMV reverse transcriptase (Promega, M510F). After 5 min at room temperature, the samples were incubated for 30 min at 37°C. The reaction was stopped by addition of 1.2 μl of 10 M NaOH and incubation for 15 min at 37°C. Subsequently, the solution was neutralized by adding 1 μl of 12 M HCl. After mixing the samples with 200 μl of a 0.3 M NaOAc solution (pH 5.5), cDNA extraction was performed with phenol/chloroform/isoamylalcohol (25/24/1). The DNA was precipitated with 3 volumes of ethanol. The pellets were briefly washed with 70% ethanol and resuspended in a formamide loading dye solution. The sequencing reactions were performed as described in (52). Finally, the cDNA was separated by denaturing 10% analytical PAGE (7 M urea), exposed to a phosphorimaging screen overnight and analyzed on a Typhoon™ FLA 9500.

### Computational identification of ambiguous start sites

To analyze the translation initiation context of genes within the *E. coli* reference genome, DAMBE software was used (53). Therefore, the *E. coli* str. K-12 substr. MG1655 reference-genome genbank file (NCBI ACCESSION: NC_000913) was downloaded and annotated coding sequences (CDSs) as well as 5′ UTRs were extracted in FASTA format. For ATGTG and GTGTG sequence motif detection, the generated FASTA files were processed and analyzed within the R-studio software suite. To import annotated genomic information the software package ‘genbankr’ was employed. Annotated pseudogenes were excluded from the analysis. To characterize initiation contexts, 5′ UTR analysis for SD-motif detection was performed in DAMBE according to (54), but only the last 9 nts of the 16S rRNA 3′ end were specified as the aSD sequence (55). To estimate expression levels of genes harboring ATGTG and GTGTG initiation sites, an integrated dataset of protein abundance was downloaded from the PaxDb database (56).

## RESULTS

The immediate vicinity of two start codons within AUGUG and GUGUG initiation sequences is intriguing as it provides two overlapping start sites for completely different ORFs. Consequently, start codon recognition must be either sufficiently precise to prevent translation of a competing ORF, or the translation of the second frame provides a regulatory or individual functional role. To better understand initiation at these peculiar sites, we characterized how initiating *E. coli* ribosomes are directed towards a defined start codon within AUGUG and GUGUG motifs, thereby providing the necessary initiation accuracy. In addition, we explored the possibility that in specific cases an ambiguous translation initiation at both start codons could occur, indicating the presence of novel ORFs hidden in the *E. coli* genome.

### AUGUG and GUGUG initiation sequences in *E*.*coli*

**Figure 1.**
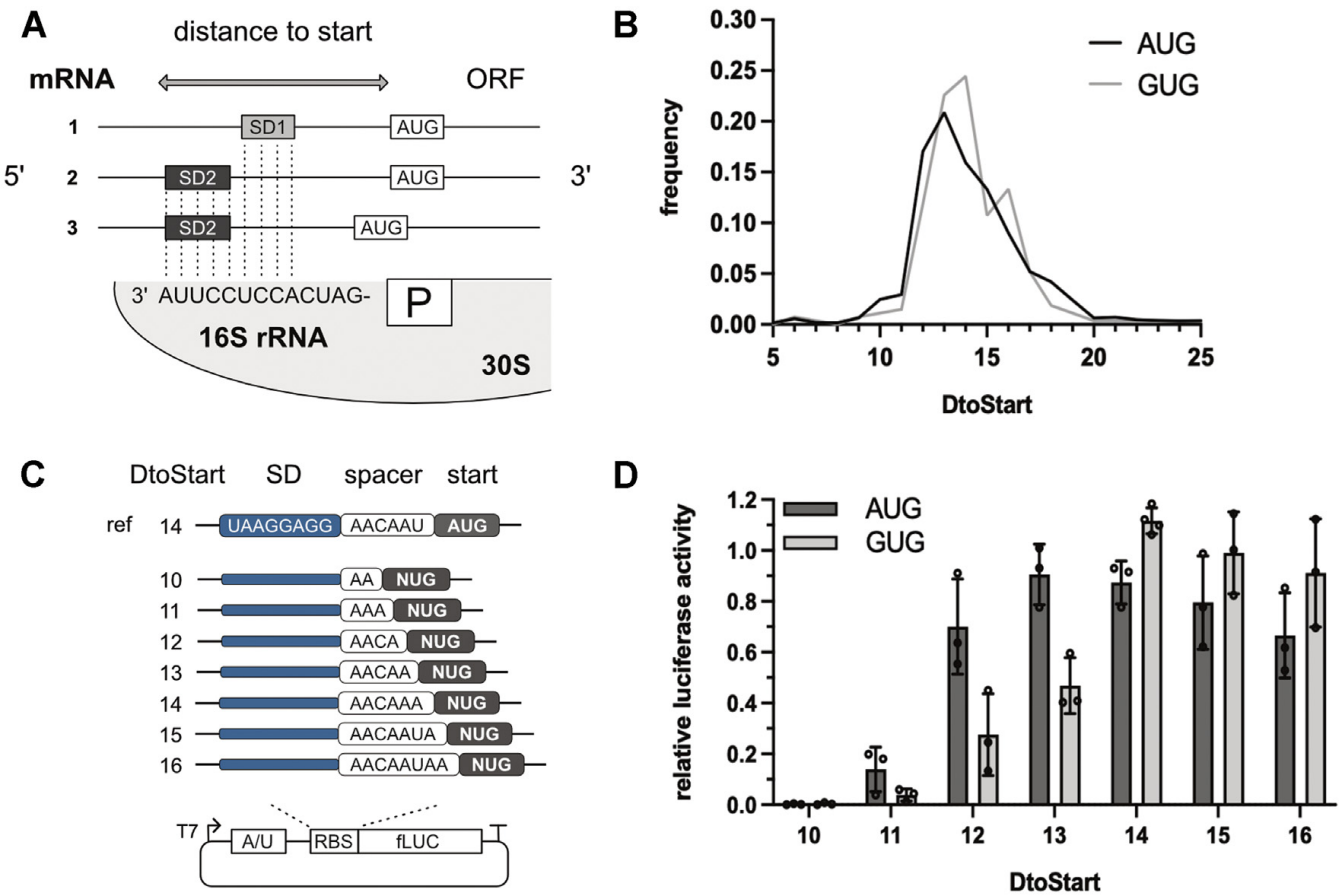
The importance of aligned spacing for initiation efficiency. (**A**) Schematic depiction of a ribosomal binding site and the SD:aSD motif interaction. Different SD motifs can position the start codon (AUG) similarly in respect to the ribosomal P-site (P). Measuring the distance from the 3′ end of the 16S rRNA to the start codon of a hybridized mRNA provides a metric for aligned spacing termed distance to start (*D*_toStart_). In the depicted example, mRNAs 1 and 2 share the same *D*_toStart_, while the aligned spacing of mRNA 3 is shorter. (**B**) Genome-wide frequency distribution of aligned spacing (*D*_toStart_) for SD-led *E. coli* genes with AUG (dark grey) or GUG (light grey) start codons. (**C**, **D**) To assess initiation efficiency in dependence of start codon identity and aligned spacing, S30 extract based luciferase reporter gene assays were carried out. (**C**) The firefly luciferase (fLUC) reporters with AUG or GUG start codons carried a strong SD motif and spacer sequences of varying lengths. The *D*_toStart_ of the luciferase genes ranged from 10 to 16 (spacer length 2 to 8 nts, respectively). (**D**) Luciferase activity depending on initiation at AUG and GUG is depicted in dark and light grey, respectively. The luminescent signal measured was related to the activity of a reference construct, carrying a distinct AUG start codon (ref). The mean and the standard deviation of the measurements are shown.

In order to identify potentially ambiguous start sites, we searched the *E. coli* reference genome for genes harboring their annotated start codons within an ATGTG or GTGTG sequence context. In total, we identified 53 genes, of which 41 accounted for ATGTG and 12 for GTGTG (Tables 1–3). Within the ATGTG sequences, 23 genes were annotated with an ATG start codon and 18 with GTG (Tables 1 and 2, respectively). For GTGTG sequences, exclusively the 3′ GTG was annotated as start codon (Table 3). As the SD sequence is a major determinant for start site recognition, we analyzed the respective 5′ UTRs of the identified genes for SD motif usage and start codon localization according to Prabhakaran et al. (54). We defined an SD sequence by any longest given stretch of four or more nts within 30 nts upstream of the CDS that potentially base pairs with the last 9 nts of the 16S rRNA 3′ end (55). To compare the positioning of the start codon overlaps in respect to the P-site between the different identified SD sequences, a metric for aligned spacing was calculated. Thus, the distance to start (*D*_toStart_) was determined, as the distance from the ultimate nucleotide of the 16S rRNA to the start codon of the aligned mRNA (Figure 1A) (54,57). Aligned spacing is known to have a profound influence on initiation efficiency and an optimal localization has been experimentally demonstrated (58–60). In the *D*_toStart_ nomenclature for aligned spacing this optimum corresponds approximately to a 14 nt distance from the 16S rRNA 3′ end. Consistently, native RBSs most frequently position their start codons at *D*_toStart_ ∼13-14 (22,54) (Figure 1B). Strikingly, our analysis revealed that most of the genes harboring ATGTG or GTGTG start sites employ an SD motif that localizes the annotated start codon closer to this optimal site than the potential competitor (Tables 1–3). This indicated that the ideal positioning likely disfavors initiation at the competing start sites, therefore ensuring an accurate initiation at the designated start codon.

**Table 1.** **ATG**tg-starting genes ranked by protein abundance (56) with their respective SD motifs (**≥**4 nts) and the aligned spacing (*D*_toStart_) to the annotated 5′ AUG start codon. Initiation sites for which ambiguity is predicted are annotated accordingly

Gene	Protein abundance (ppm)	SD sequence	*D*_toStart_	Predicted ambiguity
*ytfQ*	329.000	AGGA, GAGG	13, 16	no
*glmS*	215.000	—	—	—
*asnB*	140.000	GGAG, AGGU	13, 10	no
*yniA*	133.000	GGAG	14	no
*purF*	67.700	AGGA, GAGG	15, 18	no
*yjbQ*	26.900	AAGGAG	13	no
*trmD*	11.800	—	—	—
*yafJ*	9.410	GGAGGU	14	no
*appC*	4.720	AGGAG	12	yes
*hypB*	2.090	AGGAG	13	no
*hypC*	1.040	GGAG	15	no
*ydhB*	0.917	—	—	—
*yhdN*	0.177	GGAG	12	yes
*yedK*	0.176	AGGAGGU	13	no
*pqiA*	0.175	UAAGGAG	12	yes
*hybG*	0.094	GGAG	13	no
*ccmC*	0.045	—	—	—
*yjbM*	0.002	—	—	—
*yaaY*	—	UAAG	12	yes
*caiF*	—	GGAG	12	yes
*yobF*	—	GAGGU	21	no
*cydX*	—	UAAGGAG	13	no
*appX*	—	UAAGGAG	15	no

**Table 2.** at**GTG**-starting genes ranked by protein abundance (56) with their respective SD motifs (**≥**4 nts) and the aligned spacing (*D*_toStart_) to the annotated 5′ AUG start codon. Initiation sites for which ambiguity is predicted are annotated accordingly

Gene	Protein abundance (ppm)	SD sequence	*D*_toStart_	Predicted ambiguity
*lapA*	668.000	—	—	—
*hdhA*	447.000	AGGAGGU	13	no
*yaeP*	94.500	AGGAGG	12	no
*secF*	41.600	AGGAG	13	no
*ruvA*	28.900	AGGAG	13	no
*nsrR*	27.600	GAGGU	13	no
*wecB*	14.300	—	—	—
*tamA*	8.010	AAGGA, AGGAG	21, 12	yes
*lacI*	5.670	—	—	—
*rlhA*	3.800	UAAG	7	no
*kdpE*	3.700	GAGG	12	no
*marR*	3.320	—	—	no
*kduI*	1.250	GGAGGU	13	no
*recQ*	0.594	—	—	—
*hicA*	0.021	GGAGG	12	no
*leuE*	0.002	GAGGU	13	no
*yedR*	—	—	—	—
*napF*	—	AAGG, AGGU	10, 13	no

**Table 3.** gt**GTG**-starting genes ranked by protein abundance (56) with their respective SD motifs (**≥**4 nts) and the aligned spacing (*D*_toStart_) to the annotated 5′ AUG start codon. Initiation sites for which ambiguity is predicted are annotated accordingly

Gene	Protein abundance (ppm)	SD sequence	*D*_toStart_	Predicted ambiguity
*metH*	48.400	GGAG	12	no
*focA*	36.200	—	—	—
*nagZ*	20.300	UAAGGAG	13	no
*fes*	14.000	—	—	—
*cytR*	1.690	AGGAG	12	no
*nfrB*	0.969	UAAGG	12	no
*ycgY*	0.564	—	—	—
*yoaA*	0.204	—	—	—
*narQ*	0.115	GGAG	16	yes
*ytfT*	0.086	UAAGGAG	13	no
*yahE*	0.058	—	—	—
*pphC*	0.004	AAGG	17	yes

### Start codon selection at AUGUG and GUGUG depends on the SD motif and can result in the translation of overlapping ORFs

To experimentally address to what extent the specificity of initiation at potentially ambiguous start sites was achieved by positioning through a strong SD sequence, we employed an *in vitro* luciferase reporter assay based on *E. coli* cell extract. In a first step, distinct AUG or GUG start sites were introduced into the luciferase reporters and the *D*_toStart_ was altered from 10 to 16 by adjusting the spacer length from 2 nts to 8 nts, respectively (Figure 1C). In case of an AUG start codon, efficient initiation was observed across a broad range of positions, even at a shortened spacing of only 4 nts (*D*_toStart_ 12). At GUG, initiation showed a stronger dependence on the spacer length and especially short spacer sequences caused low initiation efficiencies. However, increasing the *D*_toStart_ to 14 and beyond provided similar luciferase activities as AUG (Figure 1D).

**Figure 2.**
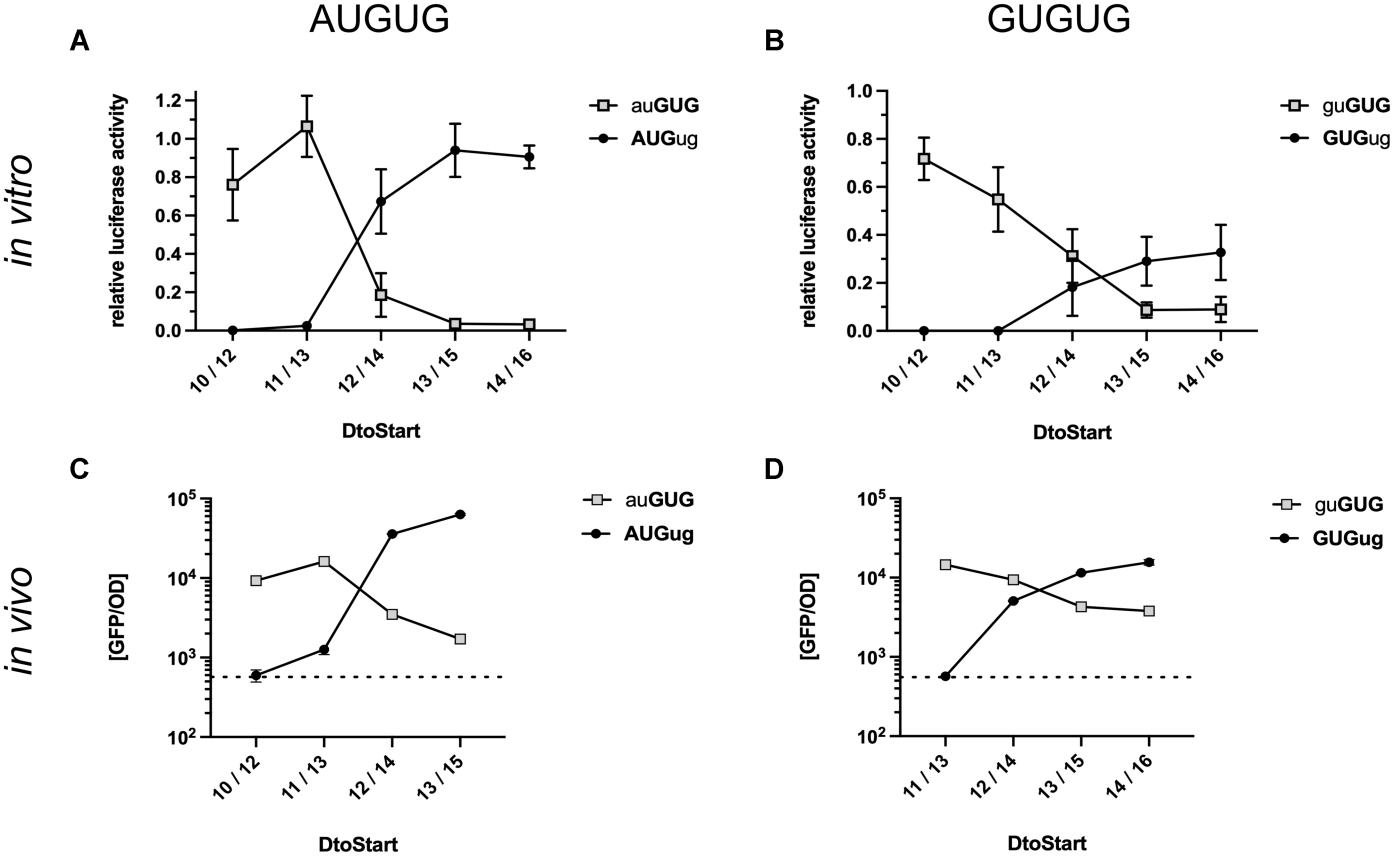
Open reading frame selection at AUGUG and GUGUG initiation sites. The reporter constructs carried a constant SD sequence (UAAGGAGG) separated from the AUGUG (**A**, **C**) or GUGUG (**B**, **D**) start codon overlaps by varying spacer lengths. The CDSs of the *in vitro* luciferase (**A**, **B**) and *in vivo* GFP (**C, D**) reporter genes were positioned in frame of either the 5′ or 3′ start codon. Expression levels depending on initiation at the 5′ start codon (black circle) and 3′ start codon (grey square) are shown as a function of the spacer length and the corresponding *D*_toStart_. The aligned spacing was varied from *D*_toStart_ 10/12 (5′/3′ start codon) to 14/16. GFP reporter constructs harbored the same SD sequence and A-rich spacer elements as the *in vitro* luciferase reporters. Measured luciferase activities were related to the activity obtained from a reference construct with a distinct AUG start site (see Figure 1A). GFP fluorescence measurements were normalized by optical density (GFP/OD_700_) and are depicted on a logarithmic scale. Cellular autofluorescent background of an uninduced sample is indicated by a dotted line. For both assays, the mean and the standard deviation of three independent measurements are shown.

As initiation at AUG and GUG was of comparable efficiency, a competition between the two start codons in an AUGUG sequence context appeared feasible. To identify a potential competition, luciferase reporters were generated harboring AUGUG start sites, in which the ORF of the luciferase was either defined by the AUG or GUG as start codon. At AUGUG start sites, a 2 or 3 nt short spacer sequence (*D*_toStart_ 10/12 and 11/13, respectively) resulted in luciferase activity exclusively from the GUG but not from the AUG start codon (Figure 2A). Conversely, employing longer spacer sequences (*D*_toStart_ 13/15 and 14/16), shifted initiation to the AUG start codon, while only little activity was measured from GUG. Strikingly, at *D*_toStart_ 12/14, activity from both ORFs was clearly detected and the activity of the AUG-starting construct was about 3.5-fold higher than GUG. These findings provided a first indication of initiation ambiguity as positioning alone could not always direct the initiation complex exclusively to AUG or GUG (Figure 2A).

In analogy to AUGUG, we also characterized start codon selection at GUGUG (Figure 2B). Once again, short spacer sequences directed the initiation complex exclusively to the 3′ GUG start codon. At *D*_toStart_ 12/14, both ORFs were translated, but unlike for AUGUG, the 3′ GUG was slightly favored. Increasing the *D*_toStart_ shifted the preference for start site selection to the 5′ GUG. Interestingly, an increased spacer length did not resolve the competition for ORF selection and even at *D*_toStart_ 14/16, both start codons were clearly used for translation initiation.

In order to confirm the initiation ambiguity at AUGUG and GUGUG sites also *in vivo*, we made use of a GFP reporter assay. GFP expression was under the control of a T5 promoter and an inducible lac operator. In analogy to the *in vitro* characterization of ambiguous start sites, the CDS of GFP was placed in frame of either start codon within the potentially ambiguous context (AUGUG and GUGUG). The same RBSs were employed for the GFP constructs as for the luciferase reporters. For the AUGUG sequence, the *D*_toStart_ varied from 10/12 to 13/15 in order to span the window, around which we had observed a shift in ORF selection. As had been the case *in vitro*, the positioning of the start codon by the SD sequence was decisive for reading frame selection *in vivo*. Consistent with our previous results, the *D*_toStart_ 12/14 resulted in GFP signals from AUG- as well as GUG-starting reading frames (Figure 2C). At *D*_toStart_ 12/14, initiation at AUG was roughly 10-fold more efficient than at GUG, compared to a 3.5-fold advantage of AUG over GUG *in vitro* (Figure 2A). We also generated reporter mRNAs harboring GUGUG, as they had appeared to be less restrictive for start codon selection. In analogy to our results from the luciferase assay, also *in vivo* the RBSs with *D*_toStart_ 12/14, 13/15 and 14/16 could not exclusively direct the initiation complex to one of the two GUG start codons but resulted in the expression of both overlapping ORFs (Figure 2D). At *D*_toStart_ 12/14, the 3′ GUG was preferentially selected over the 5′ GUG but the extension of the spacer shifted the preference to the 5′ GUG. These findings demonstrated that also under *in vivo* conditions AUGUG and GUGUG enabled the expression of both overlapping ORFs, providing ambiguous initiation sites.

**Figure 3.**
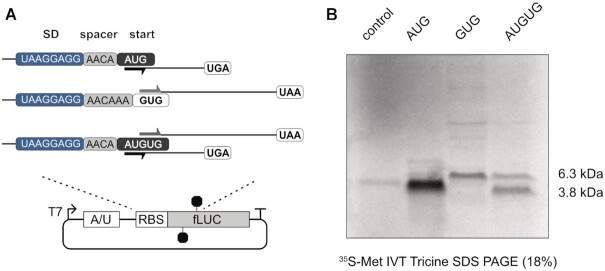
Detection of distinct peptide products by translation of two ORFs from an AUGUG ambiguous start site. (**A**) Schematic representation of the reporter plasmids for detection of translation products from either potential ORF. The RBS of the reporter sequences is depicted with the 8 nt strong SD motif (blue) and the spacer sequence (grey). (**B**) Autoradiogram of radiolabeled *in vitro* translation (IVT) products from the reporter constructs depicted in (A), including a reaction without template as negative control (control).

As so far the translation of the reporter genes had only permitted the measurement of one ORF per construct, but not the direct detection of the competing ORFs, we designed a reporter sequence encoding two differently sized peptides. Therefore, stop codons were introduced in the CDS of the luciferase reporter to create an ORF for a ∼6,3 kDa peptide in frame of GUG and a ∼3,8 kDa peptide in frame of AUG within an AUGUG initiation site (Figure 3A). The construct was then *in vitro* translated in the presence of [^35^S]-labeled Met/Cys. Indeed, both peptides from the competing ORFs could be detected, when the RBS harbored *D*_toStart_ 12/14 (Figure 3B). In respect to protein yields, the translation efficiency of the AUG ORF was about 2-3-fold higher than from the competing GUG ORF. Notably, the translation efficiencies of both overlapping ORFs were significantly lower compared to the respective controls harboring a distinct AUG or GUG start codon (Figure 3B).

### Toeprint analysis confirms ambiguous *de novo* initiation

**Figure 4.**
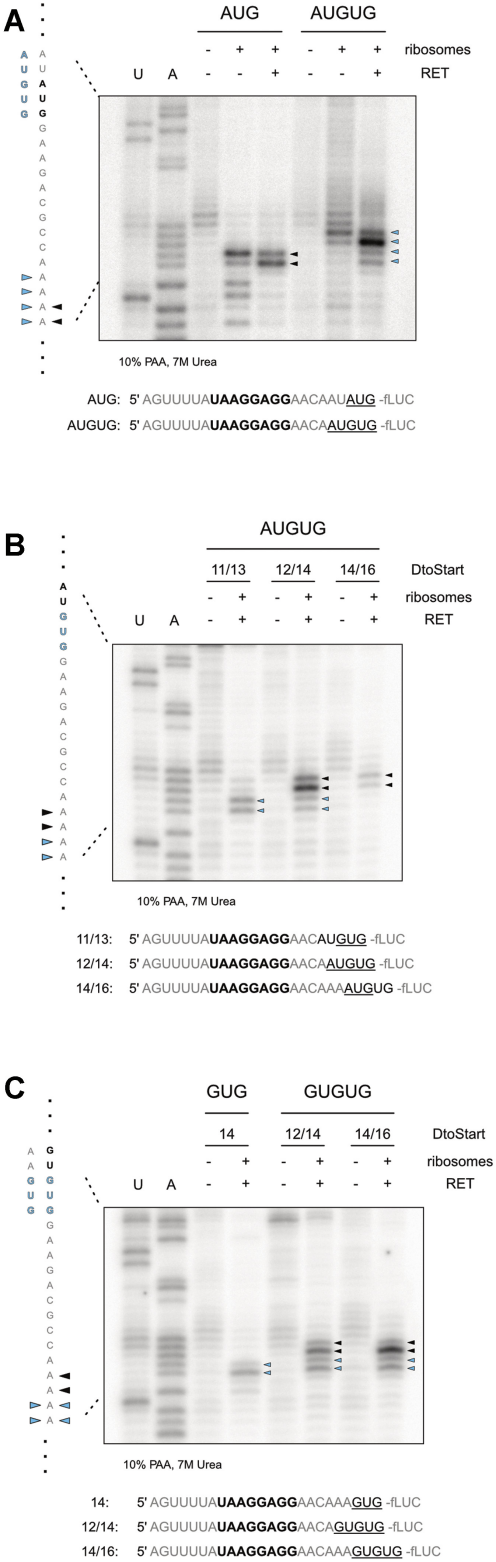
Retapamulin (RET) assisted toeprinting to determine start codon selection at AUGUG and GUGUG initiation sites. (**A**) Toeprinting analysis of fLUC reporter constructs bearing distinct (AUG) and ambiguous (AUGUG) start sites in the absence and presence of RET. Ribosome specific toeprints, at positions +16 and +17 of the CDS for mRNAs harboring a distinct AUG start codon are indicated with black arrows. At AUGUG initiation sites, toeprint signals representing two populations of initiation complexes are indicated by blue arrows. (**B**) Toeprinting analysis of start codon selection at AUGUG start sites in dependence of the positioning by a strong SD sequence. Toeprints were performed on the fLUC reporter sequences with aligned spacing of *D*_toStart_ 11/13, 12/14 and 14/16. Initiation complexes stalled at the AUG and GUG start codon are indicated by black and blue arrows, respectively. (**C**) Toeprinting analysis of start codon selection at GUGUG start sites in analogy to (B). Toeprint bands at positions +16 and +17 corresponding to the 5′ GUG start codon are indicated in black, those corresponding to the 3′ GUG in blue. Bands from the distinct GUG control, positioned as the 3′ GUG in the GUGUG sites, are indicated in blue as well. For all depicted toeprint experiments (A–C), sequencing reactions for uracil (U) and adenine (A) bases are shown with the corresponding sequence. The exact RBS context of each mRNA is provided below the respective autoradiograms. The SD sequence is indicated in bold and the selected start codons according to the toeprint patterns are underlined.

So far, our readout had been dependent on protein yields, presumably as a consequence of initiation efficiency and start codon selection. In order to directly determine the recruitment of initiation complexes to the provided start codons, toeprinting assays in the presence of the antibiotic retapamulin (RET) were performed. RET was recently demonstrated to arrest initiating ribosomes at start codons and thereby enabled the identification of novel initiation sites in *E. coli* (49). To test if RET-assisted toeprinting assays were suitable to identify ambiguity in start codon selection during *de novo* initiation, we generated mRNAs harboring distinct AUG and GUG start codons as well as AUGUG and GUGUG initiation sites. As expected, the mRNA harboring a distinct AUG start codon, resulted in two characteristic bands at positions +16 and +17 of the CDS (Figure 4A) in line with previously published results (49). When performing toeprint assays on mRNAs harboring the AUGUG initiation site, in combination with the RBS context that had provided translation of two ORFs (*D*_toStart_ of 12/14), an additional initiation site was detected (Figure 4A). The toeprints could be clearly assigned to the positions corresponding to initiation complexes stalled at AUG as well as GUG. Because RET specifically stalls ribosomes during the initiation step, the recruitment of initiation complexes to two separate start codons within the same RBS context clearly indicated actual ambiguous *de novo* initiation. Consistent with our previous experiments based on translation efficiency, the toeprints showed a more efficient initiation at AUG than at GUG. This observation markedly implied that the results obtained from the luciferase reporter assays were indeed a direct consequence of start site ambiguity at the designated competing start codons.

In order to confirm the importance of localization by the SD sequence for start codon selection, we employed mRNAs harboring different spacer lengths. In case of AUGUG, we tested *D*_toStart_ 11/13, 12/14 and 14/16, as within this range the shift in start codon selection from GUG to AUG had been clearly observed (Figure 2). Consistent with the results obtained from the reporter gene assays, a 3 nt short spacer sequence (*D*_toStart_ of 11/13) caused initiation exclusively at the GUG start codon (Figure 4B). Conversely, the extension of the spacer to 4 nts (*D*_toStart_ 12/14), resulted in toeprints at positions corresponding to both the AUG as well as the GUG start codon. Further extension of the spacer to *D*_toStart_ 14/16 directed initiation to the AUG start codon. For GUGUG ambiguous sites, start codon selection had been observed to differ from AUGUG as initiation at GUGUG seemed to be less strictly resolved by the SD sequence. A distinct GUG start site resulted in specific toeprint signals at positions +16 and +17, in analogy to a distinct AUG start codon (Figure 4C). A GUGUG start site produced an additional toeprint corresponding to the second GUG start codon (Figure 4C). Remarkably, the toeprints of the two offered start sites at *D*_toStart_ 12/14 were evenly balanced, corroborating our previous results. A clear preference for the 5′ GUG became apparent when the spacer sequence was extended to 6 nts (*D*_toStart_ 14/16). Nevertheless, the 3′ GUG was still accessible for initiation.

### Initiation at native AUGUG and GUGUG sequences reveals novel ambiguous start sites

Our initial characterization of initiation ambiguity revealed that AUGUG and GUGUG could in principle provide *de novo* initiation at both start codons. Notably, all experiments indicating start site ambiguity and positional effects had been performed in the context of a designed RBS, derived from the 5′ UTR sequence of the bacterial *ermCL* gene (48). In order to investigate initiation at start codon overlaps in naturally occurring sequence contexts, we generated luciferase reporters harboring native *E. coli* RBSs. For candidate selection, we analyzed the RBSs of ATGTG- and GTGTG-starting genes for sequence motifs that fulfilled our identified criteria for initiation ambiguity. In most cases, the annotated start codons were favorably positioned in respect to the ribosomal P-site, thereby strongly prohibiting initiation at the competing start codons (Tables 1–3). However, some candidate sequences contained a RBS, which could potentially direct initiation to both offered start codons. To test either scenario, we generated reporters that harbored 20 nts of the native 5′ UTR and 15 nts of the respective CDS, N-terminally fused to the luciferase, to provide an authentic initiation site. For all candidates the luciferase CDS was positioned in frame of either potential start codon. The exact initiation sequences are provided in the Supplementary Table S4.

**Figure 5.**
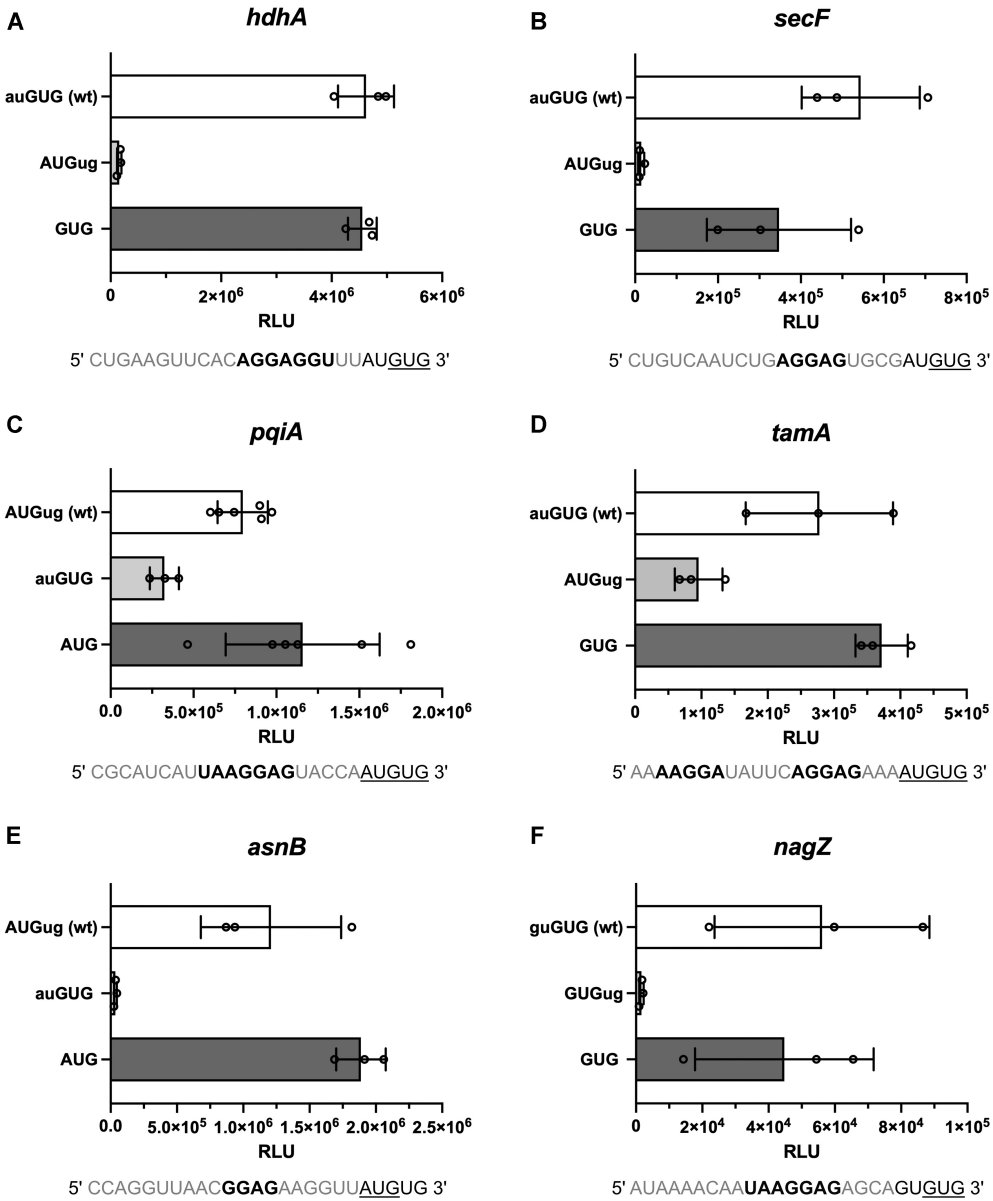
Luciferase reporter assays of native initiation sequences harboring potentially ambiguous start sites. To create an authentic initiation context, 20 nts of the native RBS and 15 nts of the corresponding CDS were fused N-terminally to the luciferase reporter CDS. Relative light units (RLU) measured from constructs harboring the fLUC CDS in frame of either potential start codon are displayed for the *E. coli* genes *hdhA* (**A**), *secF* (**B**), *pqiA* (**C**), *tamA* (**D**), *asnB* (**E**) and *nagZ* (**F**). The start codon in frame of the luciferase gene is indicated in upper case and bold. RLU measured from constructs harboring the wild type (wt) sequences are indicated in white, from the alternative ORF in light grey and from controls with distinct start codons in dark grey. The wt RBS sequences containing the AUGUG or GUGUG start sites are displayed below the respective graphs. The predicted SD sequences are depicted in bold and the determined start codons are underlined. The mean and the standard deviation of the independent measurements are provided.

The first tested candidate was *hdhA*, which carries a strong SD motif (AGGAGGU) that positions the AUGUG sequence at *D*_toStart_ 11/13, favoring the annotated GUG start codon. The length of the spacer is only 2 nts in respect to the competing AUG, therefore likely restricting initiation at this start codon. Indeed, the respective reporters provided efficient translation initiation at GUG but not at AUG (Figure 5A). Notably, initiation at GUG within the overlap was as efficient as initiation at a distinct GUG start codon, indicating that no competition between the start codons occurred.

Another candidate was *secF*, which carries an AGGAG SD sequence, localizing the AUGUG overlap at *D*_toStart_ 11/13. Whereas *hdhA* provided only a 2 nt spacer in respect to the competing AUG, *secF* harbors a 4 nt spacer to AUG, potentially allowing initiation at either start codon. However, efficient initiation was only observed at the annotated GUG start codon and hardly detectable at AUG (Figure 5B). This indicated that longer absolute spacer lengths were insufficient to compensate for the restriction imposed by short aligned spacing.

While initiation ambiguity was not observed for AUGUG candidates with *D*_toStart_ 11/13, the 5′ UTR of *pqiA* fulfilled the criteria for start site ambiguity. At *D*_toStart_ 12/14, a strong SD motif (UAAGGAG) localizes the annotated AUG start codon to potentially permit translation initiation at the competing GUG as well. Strikingly, the respective reporter mRNAs confirmed that under these premises both start codons are used for *de novo* initiation (Figure 5C). The activity from the annotated AUG start site was approximately 2-3-fold higher than from the alternative GUG-starting construct. Notably, the presence of the competing GUG start codon reduced initiation efficiency at AUG.

**Figure 6.**
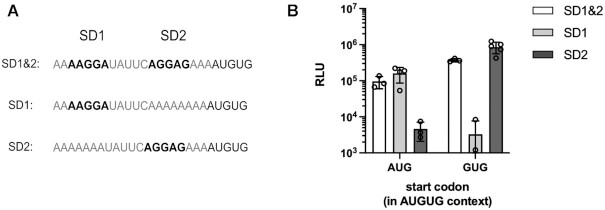
Mutational study of the *tamA* RBS to analyze the contribution of the two SD motifs to start codon selection. 20 nts of the native *tamA* RBS and 15 nts of the corresponding CDS were fused N-terminally to the luciferase reporter sequence. SD mutants were derived from the wild type sequence containing the ambiguous start site and both SD motifs (SD1 & SD2) by substituting SD1 or SD2 with a stretch of As. Replacing the start codon proximal SD motif provided the ‘SD1’ construct, replacing the upstream SD provided ‘SD2’. The tested mutant sequences of the *tamA* RBS are shown in (**A**). For each RBS variant, constructs were generated, where the luciferase CDS was placed in frame of either potential start codon. (**B**) Relative light units (RLU) measured from constructs with luciferase in frame of either AUG or GUG are displayed on a logarithmic scale for the respective RBS sequence variants. The mean and the standard deviation of the independent measurements are shown.

Whereas *pqiA* is annotated with an AUG start codon, *tamA* requires initiation at GUG within an AUGUG context. Curiously, the *tamA* 5′ UTR harbors two SD sequences of similar strength (AAGGA and AGGAG). Since these SD sequences each preferentially localize the other potential start codon, both SD motifs together could confer start site ambiguity (Figure 5D). Indeed, when testing the native *tamA* RBS, it resulted in the translation of both overlapping ORFs. The annotated GUG start codon provided 2-3-fold higher luciferase activity than the competing AUG (Figure 5D). In order to study if both SD sequences were required for the observed initiation ambiguity, we replaced each SD motif individually by a stretch of As. When eliminating the upstream SD sequence, initiation at AUG was no longer efficient, while the downstream SD directed translation to the GUG ORF (Figure 6). In contrast, the loss of the downstream SD sequence resulted in highly efficient translation of the AUG ORF, but abolished initiation at GUG. Consequently, both SD motifs directed start site selection within the ambiguous initiation context and only in concert facilitated dual translation initiation.

In analogy to *tamA*, the RBS of *asnB* did not immediately reveal a single dominant SD sequence. However, an SD motif of moderate strength (GGAG) potentially positions the AUGUG start site at *D*_toStart_ 13/15, thus favoring the annotated AUG. Consistently, initiation was only observed at AUG (Figure 5E), indicating that the moderately strong SD motif was sufficient to direct start site selection.

As GUGUG overlapping start sites had been observed to be significantly less restrictive, they were especially promising candidates for initiation ambiguity. However, most identified candidates favorably positioned the annotated 3′ GUG start codon, thereby restricting competition from the 5′ GUG by short spacing. The 5′ UTR of *nagZ*, harbors a strong SD motif (UAAGGAG) positioning its GUGUG start site at *D*_toStart_ 11/13, with a 4 nt spacer in respect to the potentially competing 5′ GUG start codon. This resulted in an exclusive initiation at the annotated 3′ GUG with no detectable translation of the competing overlapping ORF (Figure 5F).

### Adenine enrichment at the fourth CDS position consolidates start codons

Besides the SD motif, additional sequence elements have been proposed to modulate the efficiency of start codon selection. Upstream ORFs (uORFs) (61–63), A-rich sequence elements (29,42–44) as well as nucleotides in the immediate vicinity of the start codon (64–72) have been demonstrated to affect initiation efficiencies. Remarkably, in around 99% of prokaryotes an enrichment for adenine immediately downstream of the start codon (A4) was observed (73). In the *E. coli* reference genome, about half of all annotated start codons are immediately followed by A4. While beneficial effects of A4 on reporter gene expression were previously described, an underlying mechanism as well as the cause for genomic A4 enrichment have remained elusive. One potential reason could be a specific preference for certain amino acids at the N-terminus. This is contradicted by the fact that A-starting second codons appear to be highly favored over their synonymous alternatives for the 6-fold degenerate serine and arginine codons (73). Consequently, the underlying cause appears to be at the nucleotide level. In this regard, A4 could simply lower the potential for secondary structure formation and thereby improve the accessibility of the start site. Besides the structural component, A4 was also hypothesized to increase initiation efficiencies through direct interactions. One possibility could be the formation of an extended tetra-nucleotide codon/anticodon by base pairing with the conserved U at position 33 (U33) of the initiator tRNA_i_^fMet^ (71,72). Finally, A4 enrichment has also been proposed as a consequence of evolutionary selection for error-mitigation. The proposed model was based on the observation that the presence of A4 creates an immediate out of frame UGA stop codon (e.g. A**UGA**, G**UGA**) that could terminate erroneous initiation events and potentially adjust the ORF (73). The strong enrichment of A4 and its potential contribution to initiation efficiency could provide an additional determinant for start codon recognition. Because initiation ambiguity is especially challenging for start site selection, we reasoned that an ambiguous start site could be used as a tool to determine the role of A4 in start codon definition. Thus, various experiments with ambiguous start sites were performed to disentangle the potential mechanistic contributions and to put both a putative direct influence as well as the frameshift correction model to the test.

**Figure 7.**
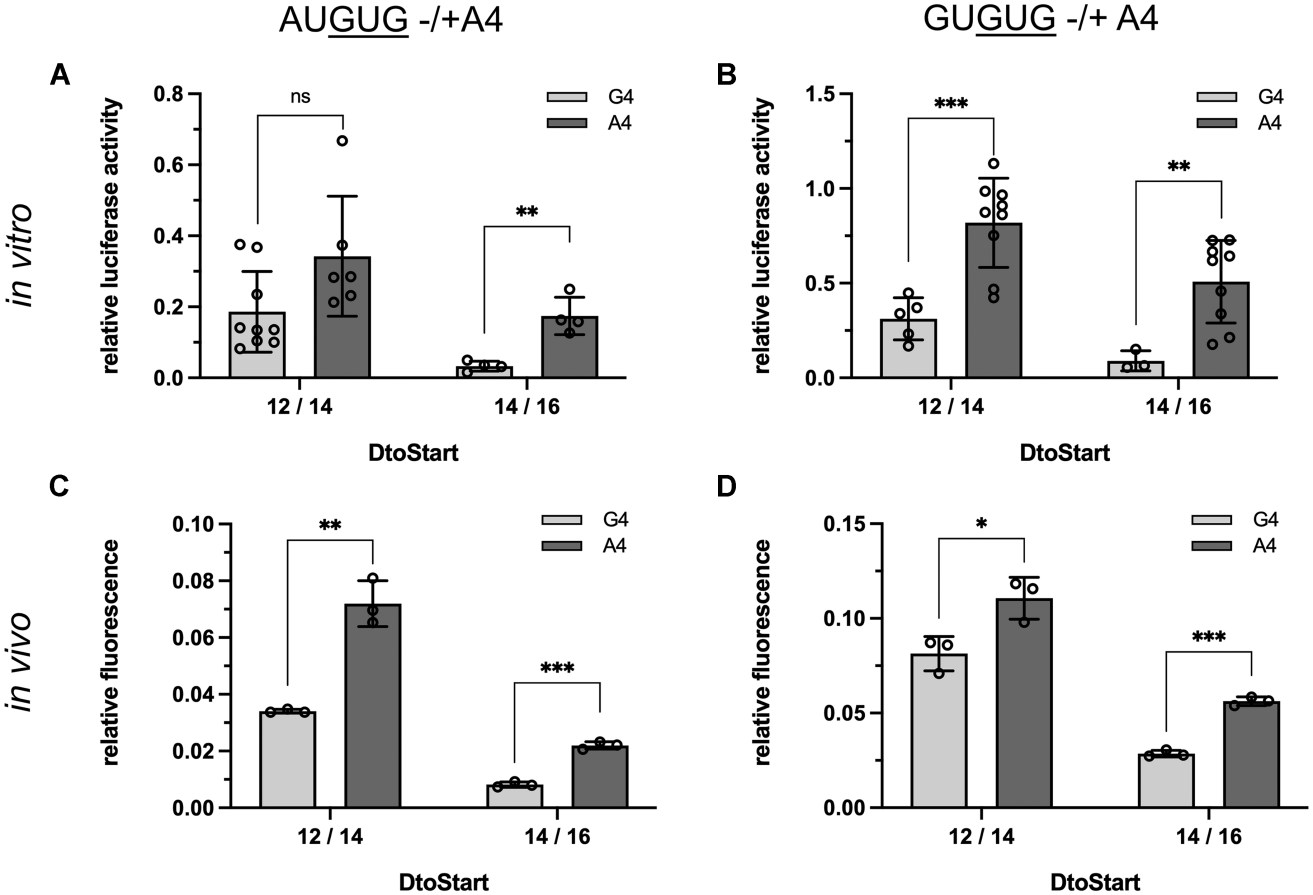
Impact of A4 on start codon selection at AUGUG and GUGUG ambiguous sites. *In vitro* luciferase (**A, B**) and *in vivo* GFP (**C, D**) reporter assays were employed to determine a potential contribution of A4 to start codon selection at AUGUG (**A**, **C**) and GUGUG (**B**, **D**) initiation sites. Reporter gene expression was dependent on initiation at the 3′ GUG start codon in frame of the respective CDS. For both reporters, constructs were generated harboring either G4 (light grey) or A4 (dark grey). The RBSs were kept constant between the luciferase and GFP reporters and the impact of A4 on initiation efficiency was monitored at two aligned spacings (*D*_toStart_ 12/14 and 14/16), providing varying degrees of initiation ambiguity. Measured relative light units from the luciferase as well as normalized GFP fluorescent signal (GFP/OD_700_) were related to the signal obtained from reference constructs harboring a distinct AUG start codon. For both the *in vitro* (A, B) and *in vivo* data (C, D), the error bars indicate the standard deviation of the independent experiments. Statistical significance was tested, employing a two-tailed, unpaired *t*-test (*P < 0.05, **P < 0.01, ***P < 0.001).

To assess whether A4 influenced start codon selection within an AUGUGA or GUGUGA context, we introduced either A4 or G4 into reporter mRNAs harboring the luciferase or GFP CDS in frame of the 3′ GUG start codon. To confer start site ambiguity, a strong SD sequence was employed, providing *D*_toStart_ 12/14. Additionally, we generated constructs, in which the aligned spacing was extended to *D*_toStart_ 14/16 to direct the initiation complex preferably to the 5′ start codon. In this experimental setup, any of the proposed contributions of A4 to start codon recognition should enhance the translation of the GUG reading frame. Indeed, the presence of A4 in the context of AUGUGA improved initiation at GUG *in vitro* as well as *in vivo* (Figure 7). Importantly, the relative impact of A4 was especially pronounced at *D*_toStart_ 14/16, when the SD sequence guided the initiation complex almost exclusively to the AUG start codon. Remarkably, this implied that either A4 was able to counteract the positional effect of the SD sequence, or the GUG-dependent reading frame became accessible only after the preceding initiation was terminated and a reinitiation step took place. In essence, the same trends were observed for GUGUGA initiation sites although the effects were not as pronounced (Figure 7B, D).

**Figure 8.**
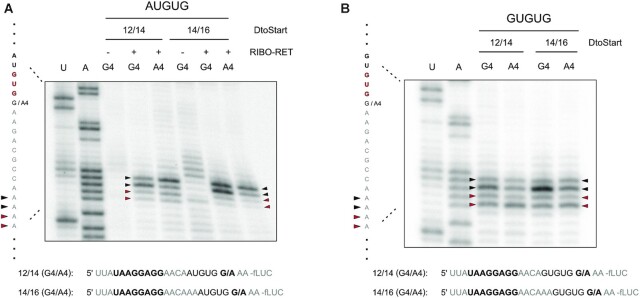
Toeprint analysis of start codon selection in absence and presence of A4. Toeprint experiments were performed on the luciferase reporter mRNAs that indicated a contribution of A4 to start codon selection (see Figure 7). A potential direct contribution of A4 to start codon selection was assessed for ambiguous AUGUG (**A**) and GUGUG (**B**) sites at the aligned spacings of *D*_toStart_ 12/14 and 14/16. Toeprints obtained by *in vitro* translation in the presence of ribosomes and retapamulin (RIBO-RET) are indicated at the positions +16 and +17 in respect to the selected initiation codon accordingly. Initiation at the 3′ GUG is indicated by red arrows, while toeprints corresponding to the 5′ start codon (AUG or GUG for A and B, respectively) are indicated by black arrows. Sequencing reactions for uracil (U) and adenine (A) bases are shown with the corresponding sequence. The exact RBS context of each mRNA is provided below the respective autoradiogram and the SD sequence as well as the fourth CDS position are indicated in bold.

To further analyze the effects of A4 on start codon selection, we carried out toeprint assays in presence of RET, which enabled us to directly determine start codon selection during *de novo* initiation. Interestingly, the toeprints did not indicate a change in the position of the initiation complex in the absence or presence of A4 (Figure 8). Even at GUGUG initiation sites, A4 did not enhance initiation at the 3′GUG (Figure 8B). These results markedly implied that A4 did not directly contribute to start codon definition via positioning of the initiation complex, even though initiation efficiencies had clearly depended on its presence (Figure 7).

### A4-dependent frameshift correction involves termination-reinitiation

The observation that A4 had the strongest impact on the reporters that effectively mimicked erroneous translation initiation indicated the involvement of a frameshift correction mechanism. In the proposed model, the termination at a UGA stop codon within a start-stop overlap (e.g. A**UGA**, G**UGA**) enables the translational machinery to adjust initiation errors towards the correct start site. The involvement of a termination-reinitiation (TeRe) event appeared to be a likely mechanistic possibility (74–76). TeRe has been postulated to facilitate translational coupling, whereby a 70S ribosome that terminates translation of an upstream ORF immediately reinitiates translation at a nearby start codon (e.g. at an overlapping A**UGA** start-stop site) (74,75).

**Figure 9.**
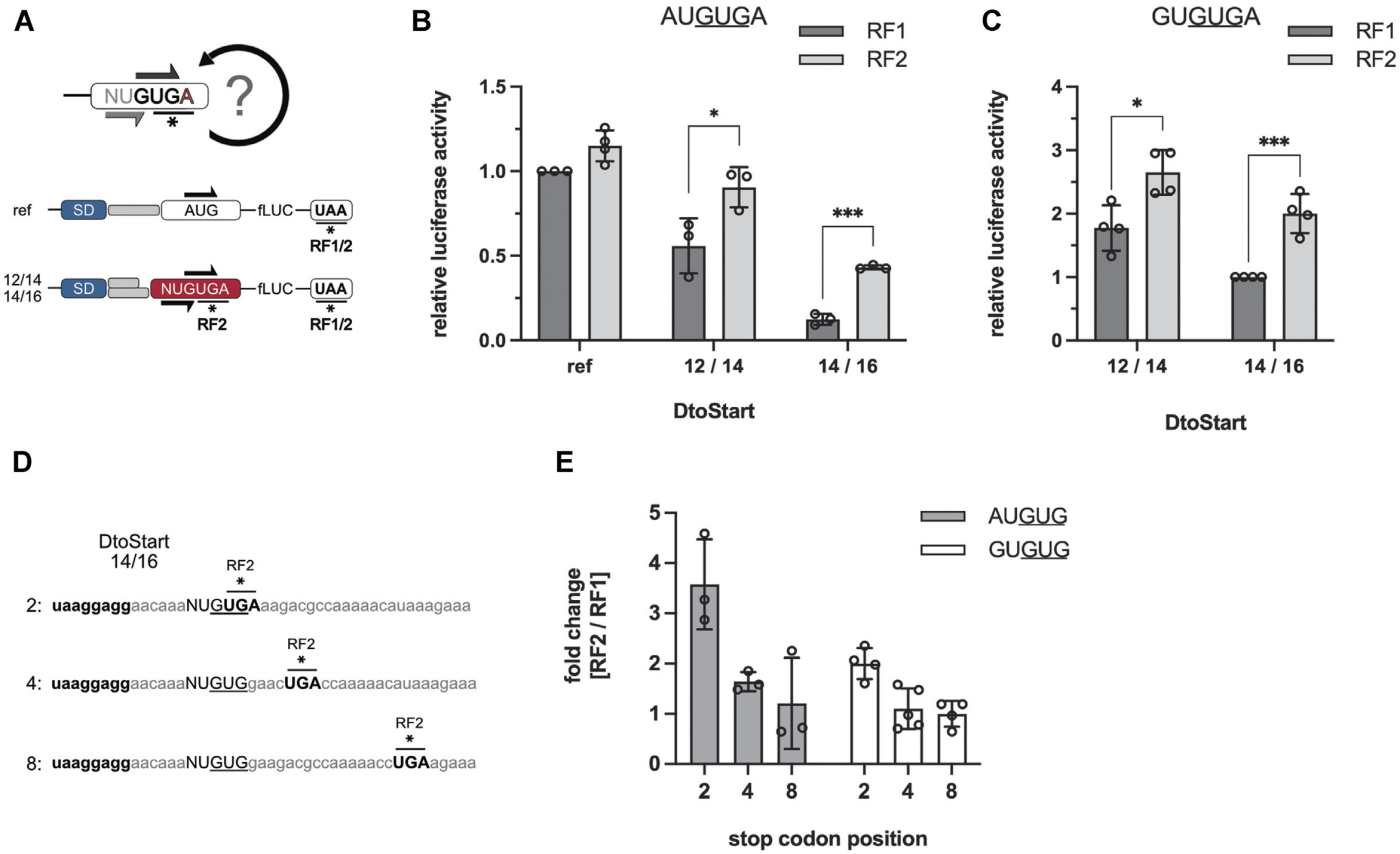
RF2-dependent activity of luciferase constructs harboring AUGUGA or GUGUGA ambiguous initiation sites. (**A**) Experimental design to determine the contribution of RF2-dependent termination to start codon selection. mRNAs with the luciferase CDS in frame of the 3′ GUG within an AUGUGA and GUGUGA context and a *D*_toStart_ of 12/14 as well as 14/16 were *in vitro* translated in presence of RF1 or RF2. As a control, a reference mRNA (ref) was employed that harbored a distinct AUG start codon and thereby depended solely on the UAA stop codon of the luciferase CDS. Relative luciferase activities of the reporter mRNAs carrying AUGUGA (**B**) or GUGUGA (**C**) initiation sites in the presence of RF1 (dark grey) or RF2 (light grey) are shown. (**D, E**) Additionally, RF2 dependence was assessed as a function of the UGA stop codon position within the 5′ start codon-dependent ORF. The initiation and termination sequence context of the employed reporter mRNAs with *D*_toStart_ 14/16 are depicted in (D). The SD motifs as well as the UGA stop codons, located at different positions within the alternative 5′ start codon-dependent ORF, are indicated in bold. The fold changes of luciferase activities in presence of RF2 compared to RF1 are depicted for AUGUG (grey) and GUGUG (white) start sites in (E). The mean and the standard deviation are shown. Statistical significance was tested, employing a two-tailed, unpaired *t*-test. (*P < 0.05, **P < 0.01, ***P < 0.001).

Because termination at UGA stop codons critically depends on the presence of release factor 2 (RF2), RF2-dependent termination would be essential for TeRe at AUG**UGA** and GUG**UGA** ambiguous start-stop sites. In order to determine whether termination was essential for the beneficial effect of A4 on initiation at ambiguous start sites, we made use of a recombinant translation system that enabled us to omit RF2. Thereby, we could directly assess the importance of an efficient termination step to translation initiation. Importantly, termination at the UAA stop codon of the luciferase CDS was still provided in the presence of RF1 (Figure 9A). In line with our previous observations, the activity of the reporter mRNA harboring *D*_toStart_ 12/14 was moderately dependent on the presence of RF2, while an increase in spacer length to *D*_toStart_ 14/16 significantly increased the RF2 dependence (Figure 9B). Therefore, termination at UGA was indeed required for efficient translation of the overlapping 3′ GUG-starting ORF. In addition to AUGUGA, we also studied the RF2 dependence of GUGUGA initiation sites, which revealed an enhanced initiation at the 3′ GUG in the presence of RF2 as well (Figure 9C). The impact of termination on the initiation at the 3′ GUG was not as pronounced as in the case of AUGUGA. This was consistent with the fact that start codon selection within GUGUG sites is not as strictly defined by positioning through the SD sequence and thereby more ribosomes initiate *de novo* at the correct 3′ GUG start codon.

Jointly, the experiments on RF2 dependence were consistent with the proposed need for an efficient termination step to facilitate a mechanism that corrects erroneous start site selection. For TeRe it has been described that reinitiation efficiency negatively correlates with the distance between the stop and the start codon (22,77). Thus, to test the proposed involvement of an immediate TeRe event, we generated mRNAs that harbored the UGA stop codon of the alternative frame further downstream of the initiation site at positions 4 and 8 (Figure 9D). If the need for an efficient termination step and thus RF2 dependence was itself dependent on the position of the UGA stop codon, then this would provide further indication of TeRe. Consistently, both for AUGUG as well as GUGUG, RF2 dependence was clearly increased when termination occurred immediately at the initiation site and declined with distance to the putative 3′ GUG reinitiation codon (Figure 9E).

### An A4-dependent ambiguous initiation site in *E*.*coli*

Because of the remarkable abundance of A4 throughout native CDSs, several of the identified AUGUG and GUGUG start codon overlaps are immediately followed by adenine, thus providing potential start-stop *de novo* initiation and subsequent TeRe sites (Tables 1–3). As previously outlined, most of the identified genes were preceded by a distinct SD sequence that efficiently directed initiation complexes to the designated start codon within the overlap. Consequently, termination in these cases might not be critically required to maintain initiation fidelity. Intriguingly, the gene *narQ* posed an exception. The annotated start codon of *narQ* is the 3′ GUG embedded within a GUGUGA context. The RBS harbors an SD sequence of moderate strength (GGAG) that provides an aligned spacing of *D*_toStart_ 14/16. According to our characterization of start site ambiguity, the 5′ GUG should be favored over the 3′ GUG, while both sites would be accessible. This implied that the translation of the *narQ* ORF could strongly depend on A4 and therefore on TeRe.

**Figure 10.**
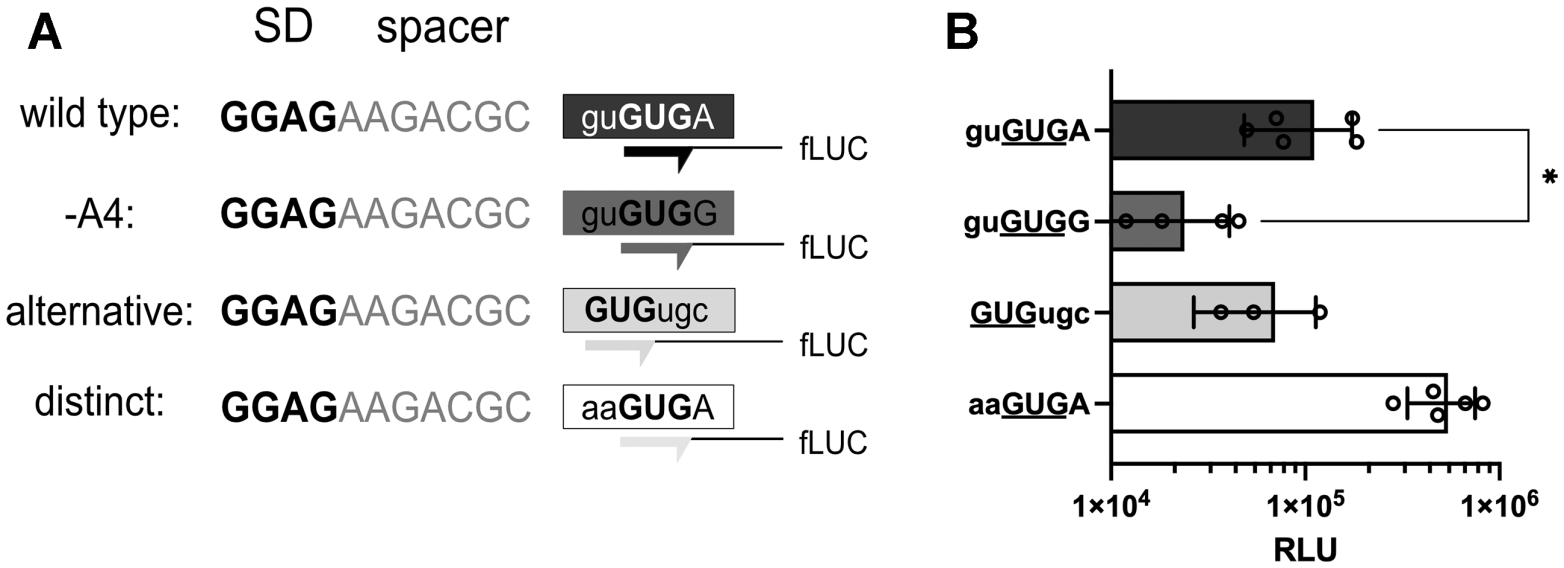
Impact of A4 on *narQ* expression. (**A**) 20 nts of the native RBS were fused N-terminally to the luciferase reporter gene, replacing its RBS to provide the GUGUGA *narQ* initiation site. The sequence of the *narQ* RBS was altered to determine the impact of A4 on the expression of the reporter mRNA. The SD motif (bold), spacer element (grey) and the initiation site (boxed) are depicted in the reporter scheme. The wild type sequence was mutated by substituting A4 with G4 (-A4). The initiation of the alternative ORF was determined by positioning the luciferase gene in frame of the 5′ GUG start codon (alternative). In order to compare the expression of the wild type sequence context to an mRNA with a distinct start codon, the ambiguous site was changed to provide only the 3′ GUG (distinct). (**B**) The measured relative light units (RLU) from the various *narQ* sequences are displayed on a logarithmic scale. RLUs of the reporters are colored according to the depiction in (A). The mean and standard deviation of the independent measurements are shown. Statistical significance was tested, employing a two-tailed, unpaired *t*-test (*P < 0.05).

To test the potential start-stop TeRe site of *narQ* and the importance of A4 in the respective sequence context, 20 nts of the *narQ* 5′ UTR, together with its GUGUGA start site, were fused to the luciferase reporter sequence. To determine the importance of A4 for the efficiency of reporter gene expression, we generated a mutant harboring G4 instead of A4 (GUGUG**G**). In additional constructs, the alternative 5′ GUG start codon was positioned in frame with the luciferase CDS within a GUGUGC context and a control harboring a distinct GUG start codon was generated (AAGUG). Indeed, both of the potential GUG start codons were used for translation initiation (Figure 10B). In the native sequence context, the annotated 3′ GUG start site provided slightly higher initiation efficiency than the competing 5′ GUG. However, the efficiency was critically dependent on A4, as initiation at GUGUGG was approximately 5-fold decreased. Strikingly, in the presence of a distinct GUG start codon, luciferase activity was increased ∼7-8-fold compared to the wild type initiation context. Jointly, these findings indicated a strong regulatory potential of the ambiguous initiation site, caused by the competition between the overlapping start codons. *narQ* represents not only an additional ambiguous initiation site in *E. coli*, but also the first identified case that depends on A4 and the described start-stop TeRe mechanism.

## DISCUSSION

Start codons within an AUGUG or GUGUG context are highly remarkable as these sequence arrangements could qualify as strong regulatory elements for gene expression or as ambiguous translation initiation sites. Even though SD motifs were recently shown to be largely dispensable for broader TIR definition (29), we hypothesized that their presence was crucial for potentially ambiguous sites, as they determine the position of start codons in respect to the ribosomal P-site. In order to characterize initiation at AUGUG and GUGUG as a function of SD-directed localization, several reporter mRNAs were created harboring strong SD sequences and spacer elements of varying length. Our results confirmed a critical role of the SD motif for start codon recognition at AUGUG and at GUGUG initiation sites. Without exception, short spacing exclusively resulted in the expression of the 3′ GUG-dependent reading frame. Extending the spacer sequences produced a shift in start codon preference, favoring the 5′ start codon. This pattern was consistent across experiments both *in vitro* and *in vivo*, independent of the reporter sequence for AUGUG as well as GUGUG (Figure 2). Therefore, start codon recognition and cistron specificity were indeed critically dependent on the localization by an SD motif.

Mechanistically, the resolution of start codon selection at instances of short and long aligned spacing appeared to be based on different principles. A strong SD motif with short aligned spacing physically prohibits the 5′ start codon from localizing near the P-site and thereby exclusively guides the initiation complex to the 3′ one. On the contrary, an extended spacing primarily increases the likelihood of localizing the 5′ start codon more favorably. This became especially apparent when considering initiation at GUGUG overlapping start sites, where no difference in base pairing potential intrinsically favored one start codon over the other. The extended spacing made both start codons accessible and both reading frames were translated. However, short spacer elements unambiguously defined the 3′ GUG as the start codon, which is reflected in the RBSs of native GTGTG-starting genes, where the annotated start codon in *E. coli* was always the 3′ one (Table 3). According to these results, only short aligned spacing provides the required specificity for efficient initiation of a single designated ORF within the GUGUG context. For AUGUG sequences, the specificity for start codon selection is also provided by the difference in base pairing free energy between AUG and GUG. At an extended spacing, initiation complexes preferred the AUG over the GUG start codon. This contribution of the codon/anticodon interaction could also be deduced from the position-dependent shift in preference for the 5′ over the 3′ start codon. Whereas for AUGUG the start codon preference shifted already between *D*_toStart_ 11/13 and 12/14, for GUGUG the shift occurred between *D*_toStart_ 12/14 and 13/15 (Figure 2).

Strikingly, the described shifts did not immediately switch exclusively from the 3′ to the 5′ start codon, but provided a window for ambiguous, dual start site selection. At AUGUG sequences, a *D*_toStart_ of 12/14 made both start codons accessible for initiation, at which AUG was favored over GUG. This preference for AUG was more pronounced *in vivo* than *in vitro*. An extended spacing beyond 12/14 drastically reduced initiation at GUG, but some residual activity was still detectable, *in vitro* as well as *in vivo*. For evenly balanced GUGUG sites, an SD motif that positions the overlap at *D*_toStart_ of 12/14 and beyond conferred initiation ambiguity (Figure 2).

In general, initiation ambiguity resulted in decreased initiation efficiencies compared to mRNAs harboring distinct start codons. Consequently, gene expression can certainly be modulated by an AUGUG or GUGUG sequence context, as previously observed (46). However, initiation efficiency was not affected if a strong SD sequence guided the initiation complex exclusively to one of the start codons. Intriguingly, the regulation by competition for start codon selection is directly linked to the translation of an alternative, overlapping ORF. While overlapping reading frames are commonly found in viral species, only few examples have been identified in bacteria, excluding terminal overlapping of genes such as AUGA start-stop overlaps (49,78,79). Especially the use of an ambiguous start site to initiate translation of overlapping reading frames is novel. To the best of our knowledge, the *rnpA-rpmH* overlap in *Thermus* represents the only described bacterial ambiguous start site so far (47). Supposedly, the overlap utilizes a single SD motif (GGAGG), which positions the two separate AUG start codons (5′ AUGGAUG 3′) in a way that permits expression of both proteins. So far, no similar instances have been reported in bacteria. In this regard, our reporter gene assays and the RET-assisted toeprints of *de novo* initiation at AUGUG and GUGUG sites, provide the first clear demonstration of ambiguous start codon selection directed by localization through a single SD motif.

As we could demonstrate that initiation ambiguity at start codon overlaps was indeed possible, we investigated the ATGTG- and GTGTG-starting genes, we had identified (Tables 1–3). Whereas in most cases the annotated start codon was ideally positioned to avoid competition, the initiation sites of *pqiA* and *tamA*, provided the capability to translate both overlapping reading frames (Figure 5). In case of *pqiA*, a single SD motif localizes the two potential start codons at *D*_toStart_ 12/14 and, as predicted, initiation at both sites could be determined. The alternative +2 frame of *pqiA* encodes a 15 amino acid long peptide (MRTSSCREAHPVLAV*), which has not yet been reported in current proteome studies of *E. coli*. It is important to note that the identification of translation products from small ORFs (smORFs) has been notoriously difficult even by mass spectrometry based approaches, limiting the detection of a likely diverse and abundant small proteome (80). Although it remains unclear from the performed *in vitro* reporter assay whether the short alternative ORF is actually expressed in *E. coli*, the principle ambiguous nature of the start site could be demonstrated. Strikingly, *pqiA* poses the first identified example of a natively encoded ambiguous initiation site that is directed by a single SD sequence, since the discovery of the *rnpA*-*rpmH* overlap in *Thermus* (47).

In contrast to *pqiA*, the *tamA* 5′ UTR harbors two SD sequences of similar strength (AAGGA and AGGAG). By mutating the *tamA* RBS and substituting either SD motif, we were able to confirm that indeed both of the two SD sequences were necessary and sufficient to provide ambiguity at the AUGUG overlap (Figure 6). While SD1 directed initiation to AUG, SD2 set the annotated GUG encoded frame. The use of the two SD motifs to facilitate dual translation initiation by *tamA* is reminiscent of the only other so far described case of initiation ambiguity, found within the *E. coli* bacteriophage lambda. There, the expression of a so-called ‘holin’ protein as well as its inhibitor ‘antiholin’, depend on the initiation at two AUG start codons in immediate vicinity (81–86). The start codons of the 105 amino acid long holin and the 107 aa long antiholin are only separated by a single lysine codon (5′ AUGAAGAUG 3′). By mutational analysis, the two SD motifs were shown to be required for initiation at either start site (81), just like we could demonstrate for *tamA*. Our results jointly indicate the expression of a 10 amino acid alternative ORF (MCAISDSYAV*) encoded by the *tamA* ambiguous start site. Importantly, additional indication for the translation of this competing reading frame has recently surfaced. In a combined analysis of ribosome profiling data for *E. coli* cells treated with antibiotics that stall ribosomes at either the start codons (RET) (49) or stop codons (apidaecin) (87), approximately 400 novel, predominantly short ORFs were identified with heightened sensitivity (88). Only the combined analysis of RET-seq and Api-seq, is suitable to determine initiation ambiguity as the use of micrococcal nuclease 1 (MNase) limits attainable resolution at the nucleotide sequence level (89,90). Remarkably, the 10 aa *tamA* alternative frame was one of the 400 newly identified smORFs (Supplementary Material; (88)). Although the alternative *tamA* ORF was not experimentally validated in our study, its detection by ribosome profiling supports our findings based on a completely different experimental approach.

With *pqiA* and *tamA* of *E. coli*, we identified two novel ambiguous initiation sites, which enable the translation of two overlapping ORFs. Whether the small peptides encoded by these short alternative frames are themselves functional and physiologically relevant, remains to be addressed by further studies. In all performed experiments, the start site ambiguity led to a decreased initiation efficiency, when compared to a distinct start site. Therefore, the translation of the alternative frame by itself may already serve a regulatory role. Importantly, the initiation at the alternative start could also unfold this regulatory potential over the main ORF in a condition-sensitive manner. The degree of initiation ambiguity could be specifically modulated in response to environmental cues, such as shifts in temperature, antibiotic stress, nutrient stress or alterations in the concentration of initiation factors. Consequently, the positional requirements for initiation ambiguity could also differ from the ones we determined under standard conditions. Especially, AUGUG sites with extended spacing could display ambiguity in a condition-dependent manner.

In conclusion, our findings not only corroborate the principle existence of an ambiguous translation initiation mechanism, but also provide the basis for future studies to reveal novel ORFs based on ambiguous start sites in a variety of bacterial species.

### Adenine enrichment at the fourth coding sequence position

Initiation at AUGUG and GUGUG sequences certainly challenges start codon recognition. Although the positioning by an SD motif can in principle provide the required accuracy for clear ORF definition, other mechanisms might come into play as well. Whereas AUGUG and GUGUG are extreme examples that can provoke erroneous ORF selection, initiation at distinct start codons could be a source for mis-initiation as well (45). Variations in the concentration of IFs or assembly defects of the ribosome could cause a reduced fidelity in start codon recognition and consequently lead to translation of non-functional ORFs (91–94). In the so-called ambush hypothesis, the evolution of out-of-frame stop codons has been postulated to mitigate frameshift/initiation errors by terminating erroneously translated ORFs and thereby preserving valuable energy and resources (95–99). It is therefore tempting to speculate that the marked enrichment of A4, across the bacterial kingdom, may have co-evolved with the NUG universal start codon to provide an N**UGA** start-stop site, immediately terminating translation of the -2 frame. The immediate overlap with the correct initiation site could provide a window of opportunity to adjust the reading frame via a proposed frameshift correction mechanism, an ideal ambush (73).

As AUGUG and GUGUG initiation sequences provide a tunable setting for ‘erroneous’ initiation, a set of experiments was designed to test the influence of A4 on gene expression and the proposed strategy for error mitigation. By employing mRNAs harboring RBSs with *D*_toStart_ 12/14 and 14/16, initiation complexes could be directed towards the ‘wrong’ 5′ start codon to varying degrees. Consistent with previous observations, A4 stimulated the reporter gene expression and therefore the initiation at the 3′ GUG start codon within the AUGUGA and GUGUGA sequence context. Remarkably, this stimulatory effect of A4 was considerably more pronounced when the initiation complexes were primarily directed towards the 5′ start codon by mRNAs carrying an RBS with *D*_toStart_ 14/16. This effect was observed *in vitro* using a luciferase reporter as well as *in vivo* employing GFP (Figure 7). It appeared unlikely that the substitution of G4 by A4 in both reporter mRNAs significantly altered the secondary structure around the start sites and thereby led to the enhanced protein expression. Furthermore, the immediate second codons that were changed to harbor A4 differed between GFP (GCA > ACA) and the luciferase mRNA (GAA > AAA), largely excluding a specific amino acid preference as a major factor for the beneficial effect. Most importantly, however, toeprinting experiments in presence of RET showed that *de novo* initiation was not significantly altered by A4 (Figure 8), excluding both putative direct interactions and structural changes around the start site as the cause. Due to these observations, the immediate termination of the erroneous -2 frame at the UGA stop codon was likely responsible for the enhanced expression instead. Consistently, omitting RF2 from *in vitro* translation reactions eliminated the beneficial effect (Figure 9A–C). Intriguingly, this increased expression due to RF2-dependent termination was critically dependent on the UGA stop codon being located immediately downstream of the ambiguous start site. Placing the UGA stop codon at codon positions 4 and 8 reduced the dependence on RF2 of the luciferase reporters that mimicked erroneous *de novo* initiation (Figure 9D, E). Jointly, these findings were most consistent with the hypothesis of a termination-reinitiation (TeRe) based mechanism that facilitated the proposed frameshift correction.

Based on these results any direct interactions with A4 that may enhance start codon recognition, such as the formation of an extended tetra-nucleotide codon/anticodon (71,72), could be largely excluded. Instead, we conjecture that A4 might have coevolved with the universal NUG start codon to facilitate efficient TeRe following erroneous *de novo* initiation events, just as polycistronic AUGA start-stop overlaps could have evolved to confer efficient translational coupling. Our experimental data is largely consistent with a previous bioinformatic analysis (73), which initially proposed A4 enrichment due to error proofing and postulated the existence of a frameshift correction mechanism to consolidate start codons.

As A4 was also frequently found following AUGUG or GUGUG initiation sites, we speculated that for some genes, the expression was modulated by ambiguous initiation and TeRe. Indeed, we identified *narQ* (initiation site GUGUGA) to strongly depend on the presence of A4. The substitution of A4 by G4 significantly reduced the expression of the reporter gene and resulted in the initiation at the competing reading frame (Figure 10). This indicated that only the proposed TeRe-dependent frameshift correction could provide the efficient translation of the *narQ* main ORF. Strikingly, the native GUGUG initiation context resulted in a strongly decreased initiation efficiency when compared to a control construct harboring a distinct GUG start codon. Thus, the peculiar initiation site of *narQ* might be an important regulatory element. Further studies are required to show if this remarkable sequence composition indeed contributes to the regulation of *narQ* expression and what benefit such a mechanism might have. In either case, the unusual initiation site of *narQ*, not only represents an additional experimentally described case of ambiguous translation initiation in *E. coli*, but also provides the first example of an initiation site that critically depends on the frameshift correction mechanism for its efficient translation.

In summary, translation initiation is a crucial step for gene expression and is therefore tightly regulated. Both the SD motif as well as A4 represent key elements of the RBS that confer start site definition and consolidate start codons and ORFs. Initiation sites harboring AUGUG or GUGUG motifs provide a novel mechanism to modulate gene expression, but also to extend the so far known ORFeome. The *E. coli* genes *pqiA*, *tamA* and *narQ* represent the first identified examples besides the aforementioned reports of the *rnpA*-*rpmH* overlap in *Thermus* and the lambda phages holin-antiholin expression. Based on our findings and the abundance of alternative start codon usage, we envision that translation initiation ambiguity is far more common than previously anticipated and expect the discovery of a multitude of ambiguous start sites in a variety of bacterial species.

## DATA AVAILABILITY

The data that support the findings of this study are available from the corresponding author upon request.

## Supplementary Material

gkac1175_Supplemental_File
