# Supplementary material for: Initiation at AUGUG and GUGUG sequences can lead to translation of overlapping reading frames in E. coli
Source: Nucleic Acids Res. 2022 Dec 22;51(1):271–89. doi: 10.1093/nar/gkac1175 (PMC9841429; doi:10.1093/nar/gkac1175)
Supplement: gkac1175_Supplemental_File [file gkac1175_supplemental_file.pdf]

Supplementary table 1*: Plasmid sequences employed for* ***figure 1*** *(The importance of aligned spacing for initiation efficiency)*

Plasmid name	Note	$D_{toStart}$	TIR (20 nt 5' UTR + 15 nt CDS)
ME_in2	distinct AUG fLUC reference	14	gtttta**taaggagg**aacaatatggaagacgccaaa
MK_in110	AUG fLUC	10	taaagtttta**taaggagg**aaatggaagacgccaaa
MK_in109	AUG fLUC	11	aaagtttta**taaggagg**aaaatggaagacgccaaa
ME_in5	AUG fLUC	12	aagtttta**taaggagg**aacaatggaagacgccaaa
ME_in4	AUG fLUC	13	agtttta**taaggagg**aacaaatggaagacgccaaa
MK_in43	AUG fLUC	14	gtttta**taaggagg**aacaaaatggaagacgccaaa
MK_in27	AUG fLUC	15	tttta**taaggagg**aacaataatggaagacgccaaa
MK_in28	AUG fLUC	16	ttta**taaggagg**aacaataaatggaagacgccaaa
MK_in112	GUG fLUC	10	taaagtttta**taaggagg**aagtggaagacgccaaa
MK_in111	GUG fLUC	11	aaagtttta**taaggagg**aaagtggaagacgccaaa
LM_05	GUG fLUC	12	aagtttta**taaggagg**aacagtggaagacgccaaa
LM_04	GUG fLUC	13	agtttta**taaggagg**aacaagtggaagacgccaaa
MK_in44	GUG fLUC	14	gtttta**taaggagg**aacaaagtggaagacgccaaa
MK_in29	GUG fLUC	15	tttta**taaggagg**aacaatagtggaagacgccaaa
MK_in30	GUG fLUC	16	ttta**taaggagg**aacaataagtggaagacgccaaa

Supplementary table 2*: Plasmid sequences employed for* ***figure 2*** *(Open reading frame selection at AUGUG and GUGUG initiation sites)*

Plasmid name	Note	$D_{toStart}$	TIR (20 nt 5' UTR + 15 nt CDS)
MK_in91	AUGug fLUC	10/12	taaagtttta**taaggagg**aaatgtgcgaagacgcc
MK_in92	AUGug fLUC	11/13	aaagtttta**taaggagg**aacatgtgcgaagacgcc
MK_in93	AUGug fLUC	12/14	aagtttta**taaggagg**aacaatgtgcgaagacgcc
MK_in94	AUGug fLUC	13/15	agtttta**taaggagg**aacaaatgtgcgaagacgcc
MK_in95	AUGug fLUC	14/16	gtttta**taaggagg**aacaaaatgtgcgaagacgcc
MK_in04	auGUG fLUC	10/12	taaagtttta**taaggagg**aaatgtggaagacgcca
MK_in03	auGUG fLUC	11/13	aaagtttta**taaggagg**aacatgtggaagacgcca
ME_in3	auGUG fLUC	12/14	aagtttta**taaggagg**aacaatgtggaagacgcca
MK_in31	auGUG fLUC	13/15	agtttta**taaggagg**aacaaatgtggaagacgcca
MK_in32	auGUG fLUC	14/16	gtttta**taaggagg**aacaaaatgtggaagacgcca
MK_in167	GUGug fLUC	10/12	taaagtttta**taaggagg**aagtgtgcgaagacgcc
MK_in168	GUGug fLUC	11/13	aaagtttta**taaggagg**aacgtgtgcgaagacgcc
MK_in156	GUGug fLUC	12/14	aagtttta**taaggagg**aacagtgtgcgaagacgcc
MK_in157	GUGug fLUC	13/15	agtttta**taaggagg**aacaagtgtgcgaagacgcc
MK_in158	GUGug fLUC	14/16	gtttta**taaggagg**aacaaagtgtgcgaagacgcc
MK_in165	guGUG fLUC	10/12	aagtttta**taaggagg**aagtgtggaagacgccaaa
MK_in166	guGUG fLUC	11/13	agtttta**taaggagg**aacgtgtggaagacgccaaa
MK_in159	guGUG fLUC	12/14	gtttta**taaggagg**aacagtgtggaagacgccaaa
MK_in160	guGUG fLUC	13/15	tttta**taaggagg**aacaagtgtggaagacgccaaa
MK_in161	guGUG fLUC	14/16	ttta**taaggagg**aacaaagtgtggaagacgccaaa
pRXG_MK16Y	AUGug GFP	10/12	taaagtttta**taaggagg**aaatgtgcgcatccaag
pRXG_MK16	AUGug GFP	11/13	aaagtttta**taaggagg**aaaatgtgcgcatccaag
pRXG_MK4	AUGug GFP	12/14	aagtttta**taaggagg**aacaatgtgcgcatccaag
pRXG_MK22	AUGug GFP	13/15	tttta**taaggagg**aacaaatgtgcgcatccaaggg
pRXG_MK17Y	auGUG GFP	10/12	aagtttta**taaggagg**aaatgtggcatccaagggc

pRXG_MK17	auGUG GFP	11/13	agtttta**taaggagg**aaaatgtggcatccaagggc
pRXG_MK3	auGUG GFP	12/14	gtttta**taaggagg**aacaatgtggcatccaagggc
pRXG_MK23	auGUG GFP	13/15	tttta**taaggagg**aacaaatgtggcatccaagggc
pRXG_MK25	GUGug GFP	11/13	aaagtttta**taaggagg**aaagtgtgcgcatccaag
pRXG_MK19	GUGug GFP	12/14	aagtttta**taaggagg**aacagtgtgcgcatccaag
pRXG_MK21	GUGug GFP	13/15	agtttta**taaggagg**aacaagtgtgcgcatccaag
pRXG_MK27	GUGug GFP	14/16	gtttta**taaggagg**aacaaagtgtgcgcatccaag
pRXG_MK24	guGUG GFP	11/13	agtttta**taaggagg**aaagtgtggcatccaagggc
pRXG_MK12	guGUG GFP	12/14	gtttta**taaggagg**aacagtgtggcatccaagggc
pRXG_MK20	guGUG GFP	13/15	tttta**taaggagg**aacaagtgtggcatccaagggc
pRXG_MK6	guGUG GFP	14/16	ttta**taaggagg**aacaaagtgtggcatccaagggc

***Supplementary table 3**: Plasmid sequences employed for **figure 3** (Detection of distinct peptide products by translation of two ORFs from an AUGUG ambiguous start site)*

**Plasmid name**	**Note**	**Dt_{oStart}**	**TIR**
MK_in136	3.8 kDa AUG ORF	12	aagtttta**taaggagg**aacaatgaagaagacgcca
MK_in137	6.3 kDa GUG ORF	14	aagtttta**taaggagg**aacaaagtggaagacgcca
MK_in126	3.8 & 6.3 kDa AUGUG ORFs	12/14	aagtttta**taaggagg**aacaatgtggaagacgcca

***Supplementary table 4**: Plasmid sequences employed for **figure 5** (Luciferase reporter assays of native initiation sequences harboring potentially ambiguous start sites)*

Plasmid name	Note	$D_{toStart}$	Native TIR - fLUC fusion
MK_in117	*hdhA* auGUG (wt) fLUC	11/13	gaagttcac**aggaggt**ttatgtgtttaattctgacgaagac
MK_in119	*hdhA* AUGug fLUC	11/13	gaagttcac**aggaggt**ttatgtgctttaattctgacgaagac
MK_in118	*hdhA* GUG control fLUC	13	gaagttcac**aggaggt**ttacgtgtttaattctgacgaagac
MK_in176	*secF* auGUG (wt) fLUC	11/13	ctgtcaatctg**aggag**tgcgatgtggcacaggaatatgaagac
MK_in177	*secF* AUGug fLUC	11/13	ctgtcaatctg**aggag**tgcgatgtggcacaggaatgaagac
MK_in178	*secF* GUG control fLUC	13	ctgtcaatctg**aggag**tgcgaagtggcacaggaatatgaagac
MK_in107	*pqiA* AUGug (wt) fLUC	12/14	cgcatcat**taaggag**taccaatgtgcgaacatcatgaagac
MK_in108	*pqiA* auGUG fLUC	12/14	cgcatcat**taaggag**taccaatgtgcgaacatcatcagaagac
MK_in130	*pqiA* AUG control fLUC	12	cgcatcat**taaggag**taccaatgagcgaacatcatgaagac
MK_in179	*tamA* auGUG (wt) fLUC	19/21; 10/12	aaa**aagga**tattc**aggag**aaaatgtgcgctatatccgagaagac
MK_in180	*tamA* AUGug fLUC	19/21; 10/12	aaa**aagga**tattc**aggag**aaaatgtgcgctatatccgaagac
MK_in181	*tamA* GUG control fLUC	21; 12	aaa**aagga**tattc**aggag**aaaaagtgcgctatatccgagaagac
MK_in185	*asnB* AUGug (wt) fLUC	13/15	accaggttaac**ggag**aaggttatgtgttcaattttttgaagac
MK_in186	*asnB* auGUG fLUC	13/15	accaggttaac**ggag**aaggttatgtgttcaattttgaagac
MK_in187	*asnB* AUG control fLUC	13	accaggttaac**ggag**aaggttatgagttcaattttttgaagac
MK_in182	*nagZ* guGUG (wt) fLUC	11/13	aataaaacaa**taaggag**agcagtgtgggtccagtaatggaagac
MK_in183	*nagZ* GUGug fLUC	11/13	aataaaacaa**taaggag**agcagtgtgggtccagtacgaagac
MK_in184	*nagZ* GUG control fLUC	13	aataaaacaa**taaggag**agcaaagtgggtccagtaatggaagac

Supplementary table 5*: Plasmid sequences employed for* ***figure 6*** *(Mutational study of the tamA RBS to analyze the contribution of the two SD motifs to start codon selection)*

**Plasmid name**	**Note**	**$D_{toStart}$**	**Native TIR - fLUC fusion**
MK_in179	tamA (SD1&2) auGUG fLUC	19/21; 10/12	aaa**aagga**tattc**aggag**aaaatgtgcgctatatccgagaagac
MK_in180	tamA (SD1&2) AUGug fLUC	19/21; 10/12	aaa**aagga**tattc**aggag**aaaatgtgcgctatatccgaagac
MK_in210	tamA (SD1) auGUG fLUC	19/21	aaa**aagga**tattcaaaaaaaaatgtgcgctatatccgagaagac
MK_in213	tamA (SD1) AUGug fLUC	19/21	aaa**aagga**tattcaaaaaaaaatgtgcgctatatccgaagac
MK_in209	tamA (SD2) auGUG fLUC	10/12	aaaaaaaatattc**aggag**aaaatgtgcgctatatccgagaagac
MK_in212	tamA (SD2) AUGug fLUC	10/12	aaaaaaaatattc**aggag**aaaatgtgcgctatatccgaagac

Supplementary table 6*: Plasmid sequences employed for* ***figure 7*** *(Impact of A4 on start codon selection at AUGUG and GUGUG ambiguous sites)*

**Plasmid name**	**Note**	**$D_{toStart}$**	**TIR (20 nt 5' UTR + 15 nt CDS)**
ME_in2	distinct AUG fLUC reference	14	gtttta**taaggagg**aacaatatggaagacgccaaa
ME_in3	auGUG fLUC	12/14	gtttta**taaggagg**aacaatgtggaagacgccaaa
MK_in06	auGUGA fLUC	12/14	gtttta**taaggagg**aacaatgtgaaagacgccaaa
MK_in32	auGUG fLUC	14/16	ttta**taaggagg**aacaaaatgtggaagacgccaaa
MK_in34	auGUGA fLUC	14/16	ttta**taaggagg**aacaaaatgtgaaagacgccaaa
MK_in159	guGUG fLUC	12/14	gtttta**taaggagg**aacagtgtggaagacgccaaa
MK_in162	guGUGA fLUC	12/14	gtttta**taaggagg**aacagtgtgaaagacgccaaa
MK_in161	guGUG fLUC	14/16	ttta**taaggagg**aacaaagtgtggaagacgccaaa
MK_in164	guGUGA fLUC	14/16	ttta**taaggagg**aacaaagtgtgaaagacgccaaa
pRXG_MK1	distinct AUG GFP reference	14	gtttta**taaggagg**aacaatatggcatccaagggc
pRXG_MK3	auGUG GFP	12/14	gtttta**taaggagg**aacaatgtggcatccaagggc
pRXG_MK5	auGUGA GFP	12/14	gtttta**taaggagg**aacaatgtgacatccaagggc
pRXG_MK6	auGUG GFP	14/16	ttta**taaggagg**aacaaaatgtggcatccaagggc
pRXG_MK7	auGUGA GFP	14/16	ttta**taaggagg**aacaaaatgtgacatccaagggc
pRXG_MK12	guGUG GFP	12/14	gtttta**taaggagg**aacagtgtggcatccaagggc
pRXG_MK14	guGUGA GFP	12/14	gtttta**taaggagg**aacagtgtgacatccaagggc
pRXG_MK13	guGUG GFP	14/16	ttta**taaggagg**aacaaagtgtggcatccaagggc
pRXG_MK15	guGUGA GFP	14/16	ttta**taaggagg**aacaaagtgtgacatccaagggc

Supplementary table 7*: Plasmid sequences employed for* ***figure 9*** *(RF2 dependent activity of luciferase constructs harboring AUGUGA or GUGUGA ambiguous initiation sites)*

Plasmid name	Note	$D_{toStart}$	RBS and leading CDS with alternative frame termination site
MK_in06	auGUGA fLUC	12/14	gtttta**taaggagg**aacaatg**TGA**aagacgccaaa
MK_in34	auGUGA fLUC	14/16	ttta**taaggagg**aacaaaatg**TGA**aagacgccaaa
MK_in162	guGUGA fLUC	12/14	gtttta**taaggagg**aacagtg**TGA**aagacgccaaa
MK_in164	guGUGA fLUC	14/16	ttta**taaggagg**aacaaagtg**TGA**aagacgccaaa
MK_in34	auGUGA (2nd codon stop) fLUC	14/16	**taaggagg**aacaaaatg**TGA**aagacgccaaaaacataaagaaa
MK_in68	auGUG (4th codon stop CUGA) fLUC	14/16	**taaggagg**aacaAAatgtggaac**TGA**ccaaaaacataaagaaa
MK_in97	auGUG (8th codon stop CUGA) fLUC	14/16	**taaggagg**aacaaaatgtggaagacgccaaaaacc**TGA**agaaa
MK_in164	guGUGA (2nd codon stop) fLUC	14/16	**taaggagg**aacaaagtg**TGA**aagacgccaaaaacataaagaaa
MK_in171	guGUG (4th codon stop CUGA) fLUC	14/16	**taaggagg**aacaaagtgtggaac**TGA**ccaaaaacataaagaaa
MK_in172	guGUG (8th codon stop CUGA) fLUC	14/16	**taaggagg**aacaaagtgtggaagacgccaaaaacc**TGA**agaaa

Supplementary table 8*: Plasmid sequences employed for* ***figure 10*** *(Impact of A4 on narQ expression)*

Plasmid name	Note	$D_{toStart}$	*narQ* native TIR - fLUC fusion
MK_in202	*narQ* +A4 (wt) fLUC	14/16	attttttgt**ggag**aagacgcgtgtgaaagacgccaaa
MK_in203	*narQ* -A4 fLUC	14/16	attttttgt**ggag**aagacgcgtgtggaagacgccaaa
MK_in204	*narQ* alt frame fLUC	14/16	attttttgt**ggag**aagacgcgtgtgcaaagacgccaaa
MK_in205	*narQ* distinct GUG fLUC	14/16	attttttgt**ggag**aagacgcaagtgaaagacgccaaa
